# Relationship between neonatal glutamine supplementation and amino acid profiles, protein synthesis in splanchnic tissues and the plasma metabolome in 5-day-old piglets with low birth weight

**DOI:** 10.1371/journal.pone.0359788

**Published:** 2026-10-12

**Authors:** Miriama Sciascia, Johannes Buchallik-Schregel, Zeyang Li, Solvig Görs, Armin Tuchscherer, Andreas Hoeflich, Ann-Kristin Henning, Jürgen Zentek, Cornelia C. Metges

**Affiliations:** 1 Nutritional Physiology, Research Institute for Farm Animal Biology (FBN), Dummerstorf, Germany; 2 Statistics in Genomics, Research Institute for Farm Animal Biology (FBN), Dummerstorf, Germany; 3 Endocrinology of Farm Animals, Research Institute for Farm Animal Biology (FBN), Dummerstorf, Germany; 4 Institute of Microbiology, University of Greifswald, Greifswald, Germany; 5 Institute of Animal Nutrition, Department of Veterinary Medicine, Free University of Berlin, Berlin, Germany; Institute of Biomedicine of Seville-IBiS, SPAIN

## Abstract

The incidence of low birthweight (**LBiW**) piglets, which show impaired growth compared to normal birthweight (**NBiW**) littermates, has increased with litter size. We previously reported that glutamine increased the bodyweight and plasma glutamine concentrations in LBiW piglets, with no effect on jejunal glutamine concentrations or glutamine transport and metabolism mRNA abundance. In this study, we investigated the effect of glutamine supplementation on proteinogenic free amino acid (**FAA**), protein-bound AA (**PBAA**) and AA-metabolite profiles, mRNA abundance, fractional protein synthesis (**FPSR**) and the plasma metabolome. At birth (0 days of age (**d**)), 24 pairs of male LBiW (1.10 kg ± 0.02) and NBiW (1.50 kg ± 0.02) littermates were selected. At 1 d, litters were standardized to 12 piglets, and piglets supplemented daily with either glutamine (1 g/kg bodyweight) or isonitrogenous amounts of alanine (1.22 g/kg bodyweight), until 5 d. Stomach content FAA, AA-metabolites, duodenal and liver tissue FAA, PBAA, AA-metabolites, biochemical indices, duodenal, colon and liver tissue FPSR, liver tissue and duodenal mucosa glutamine transport and metabolism mRNA abundance and the plasma metabolome were analysed. Stomach digesta glutamine (*P* < 0.001) and glutamate (LBiW, *P* = 0.02; NBiW, *P* = 0.03) concentrations were higher in glutamine than alanine piglets. Analysis by Data Integration Analysis for Biomarker Discovery using Latent Variable Approaches for Omics Studies showed glutamine, carnosine, citrulline and alanine separated the supplementation groups, whilst glutathione reductase, glutathione synthetase and tight junction protein 1 mRNA separated glutamine LBiW piglets from alanine. Duodenal RNA was lower and Protein-to-RNA ratio higher (*P* = 0.004) in glutamine than alanine LBiW piglets (*P* = 0.02). Colon FPSR was higher in NBiW than LBiW piglets supplemented with Ala. No effects on the plasma metabolome were observed. These results show that not all glutamine passed through the stomach, and that glutamine is absorbed and metabolised in the duodenum, potentially altering the intracellular pH, and appears to increase oxidative stress markers in the duodenum of LBiW piglets.

## Introduction

Increasing sow prolificacy to meet productivity demands has led to an increased incidence of low birthweight (**LBiW**) piglets [1]. Low birthweight piglets exhibit higher mortality rates, impaired growth and organ development, compared with normal birthweight (**NBiW**) piglets [1], which is associated with economic losses and compromised animal welfare. Researchers are therefore seeking ways to improve growth and organ development in LBiW piglets. Supplementation studies with the amino acids (**AA**) glycine [2] and arginine [3,4] have been reported in piglets, and one of the most studied AA is glutamine (**Gln**) [5,6].

Studies on Gln supplementation in pregnant sows have shown a positive effect on litter weight, a lower incidence of LBiW piglets [7,8], as well as increased the small intestinal weight and abundance of extracellular matrix and tight junction proteins in LBiW piglets [8]. Whilst supplementation studies during the suckling phase have reported that when Gln is given to the sows, it leads to increased milk production, altered milk composition, improved piglet average daily gain and weaning bodyweight (**BW**) [9]. However, no specific effects on LBiW piglet performance have been reported in studies of sows supplemented with Gln during lactation. We have previously reported that direct supplementation of Gln to suckling piglets improved LBiW piglet BW, during [10,11] and post [12] supplementation, altered the plasma profile of some metabolites, increased plasma Gln concentrations and was associated with reduced colonic concentrations of biogenic amines, compared to LBiW piglets supplemented with alanine. However, no differences in tissue jejunal Gln concentrations, fractional protein synthesis, or the mRNA abundance of genes involved in Gln transport or metabolism were observed.

We formulated the following hypotheses: (1) not all of the supplemental Gln had transited out of the stomach within the observation period, (2) glutamine was mainly absorbed before the mid-jejunum, (3) the supplemental amount of Gln changed the AA profiles of the duodenal tissue and liver as well as the abundance of mRNA transcripts associated with Gln transport and metabolism, and the duodenal and colon tissue and liver fractional protein synthesis, and (4) changes occur in the plasma metabolome reflecting whole body AA metabolism. Therefore, in the present study we supplemented Gln to suckling piglets during the first 5 days of life, and subsequently examined the AA profiles in the stomach content, duodenal tissue and liver, as well as the mRNA abundance of genes involved in Gln transport and metabolism in the duodenal mucosa and liver, and the plasma metabolome.

## Materials and methods

### Ethics statement

This animal study was performed between October 2016 and January 2018, at the Research Institute for Farm Animal Biology’s (**FBN**) experimental pig facility and all data has been reported as per the ARRIVE 2.0 guidelines. Ethical approval for all animal experimental procedures was granted by the State Office for Agriculture, Food Safety and Fishery, Mecklenburg-Western Pomerania, Germany (permission No. 7221.3-1-026/16), and conducted according to the German Animal Welfare Act (Directive 2010/63/EU, protection of animals used for scientific purposes).

### Sow housing conditions and diets

The insemination, pregnancy confirmation and farrowing induction methods and housing conditions of the German Landrace gilts used in this study, have all been described in detail in a previous paper [13]. Gilts were fed standard pregnancy and lactation diets (pregnancy: 11.4 MJ of metabolizable energy/kg, 12.6% crude protein, 3.8% ether extract, 9% fiber; lactation: 13.2 MJ of metabolizable energy/kg, 16.5% crude protein, 6% ether extract, 5.3% fiber; Trede & v. Pein, Germany), following German energy and nutrient recommendations for pregnant and lactating sows [14].

### Experimental litter and piglet selection

For this study, litters were selected based on the following parameters: (1) the number of live and stillborn piglets (excluding mummies) per litter ranged from 12 and 20, and (2) each litter contained at least one pair of male littermates, one of whom had a low birthweight (**BiW**) (LBiW; 0.8 to 1.2 kg; below the lowest quartile of piglet BiW at the FBN pig facility) and one with a normal BiW (NBiW; 1.40 to 1.80 kg; within the middle 50^th^ percentile), and (3) the farrowing duration, defined as the difference between the first and last piglet, did not exceed 6 hours (**h**). The litter size range was chosen to minimise the effect of litter on growth and survival within the first 24 h of life. Male pure German Landrace piglets were selected as they are reported to have a higher pre-weaning mortality than females [15], and we hypothesized that they would benefit more from additional dietary Gln supply. Furthermore, the study design was intended to exclude the effect of piglets’ sex and any potential negative effect of prolonged farrowing times on piglet survival [16,17]. The exclusion criteria for the study were published in a companion study [10] and based on these criteria, five LBiW and one NBiW piglet were excluded from the experiment and replaced with matching BiW piglets to achieve the required sample size (48 piglets). Therefore, for each of the LBiW (1.10 kg ± 0.02) and NBiW (1.50 kg ± 0.02) groups, 12 pairs of male littermates were selected, born to 16 gilts spread across 8 farrowing dates, from litters of 14–18 piglets with a mean farrowing time of 251 min ± 0:02 (**S1 Table**). All selected litters were standardised to 12 piglets 24 h post-farrowing and included 1–3 pairs of experimental piglets, which remained with their birth dams throughout the study.

### Study design

At 24 h after birth LBiW and NBiW piglets were assigned to Gln (*n* = 24) or Alanine (**Ala**; *n* = 24) supplementation groups (**Suppl**). The piglets were allocated to the individual Suppl groups in such a way that there was no significant difference in BW between piglets of the same BiW group. This resulted in four experimental groups (*n* = 12/group) with a mean BiW of LBiW-Gln (1.17 kg ± 0.03), LBiW-Ala (1.11 kg ± 0.03) and NBiW-Gln (1.55 kg ± 0.04), NBiW-Ala (1.56 kg ± 0.04). Randomisation was not used to assign piglets to Gln or Ala supplementation groups. The AA supplementation protocol has been described in a previous study [10], and was designed so that Gln supplemented piglets received 1 g/kg BW/day, and Ala controls were given an amount isonitrogenous (1.22 g/kg BW/day) to the Gln dose. The amount of supplemental Gln was selected based on a study which reported improved average daily gain (**ADG**) of 7 d of age (d) suckling piglets supplemented for one week, compared to Ala or water supplemented controls [18]. Experimental staff were not blinded to the supplementation. At 5 d, piglets were transported in pairs to the FBN slaughterhouse (duration approximately 5 min), to ensure that they were not alone during transport or in the holding pen, in order to reduce stress. In the holding pen, piglets were provided with straw bedding, a heating lamp, and free access to milk replacer (150 g/l water; 20·5% CP, 10% EE, 0·2% fibre; Neopigg Rescuemilk 2·0, Provimi). Two h prior to euthanasia, piglets were given 33% of their daily AA supplement in 6 mL milk replacer, and remained in the holding pen until sacrificed. Euthanasia was carried out in accordance with the German Ordinance on the Protection of Animals during Slaughter or Euthanasia, implementing Regulation (EC) No 1099/2009 of the European Council (Ordinance on the Protection of Animals during Slaughter – TierSchlV). A physical method was used to kill the pigs, as chemical residues of anaesthetics in the plasma would render subsequent laboratory analyses unusable. The animals were stunned using a small captive bolt pistol, which causes the immediate destruction of the relevant areas of the brain. Immediately afterwards, an incision was made through the carotid artery, and the piglets were bled out whilst in a state of deep unconsciousness. Blood samples were collected into K-EDTA tubes (Sarstedt, Germany), and centrifuged for 20 min at 1576 × *g* and 4 °C to obtain plasma. The plasma was then stored at −80 °C for subsequent targeted metabolomics analysis. The intestine was clamped at the junction of the pylorus and the duodenum, to allow for collection of the stomach content, and at the duodenojejunal flexure, to enable sampling of whole duodenal tissue and duodenal mucosa. The duodenal mucosa was obtained by scraping of the mucosal layer using a glass microscope slide as previously described [19]. Liver tissue was sampled between the right and left medial lobes, avoiding veins. All tissue samples were collected within 5 min of euthanasia, snap-frozen in liquid N_2_, and stored at −80 °C for subsequent analysis.

### Piglet growth and litter characteristics

On the day of farrowing (0 d), piglet sex, BiW, birth time (h:min) and 4 h after birth BW were recorded and percentage BW gain from birth to 4 h after birth was calculated as follows:


$$\begin{array}{c} Percentage\;BW\;gain=\frac{\left(\text{4}\;h\;after\;birth\;BW-\;BiW\right)}{BiW}*\text{1}\text{0}\text{0} \end{array}$$

Litter characteristics – number of live and stillborn as well as mummies, the duration of farrowing (time to the birth of the first and last piglet (h:min)) were recorded, and intra-litter variability were calculated [20] (S1 Table). Piglet BW, abdominal circumference, crown-rump length, rectal temperature, body mass index and ponderal index for the entire experimental period, and time to suckle were recorded in a companion study [10].

### Plasma clinical parameters, immunoglobulins, IGF1 and IGF-binding proteins

At 4 h after birth, a sub-population of piglets (*n* = 16 per BiW group) were placed on a sampling table in a supine position, and 0.5 mL of blood was collected by anterior vena cava puncture, with the entire procedure lasting from 5–10 s per piglet. Blood samples were collected into K-EDTA tubes (Sarstedt, Germany), and centrifuged for 20 min at 1576 × *g* and 4 °C to obtain plasma. The plasma was then stored at −80 °C for subsequent analysis. The concentrations of albumin, bilirubin, cholesterol, glucose, urea, lactate, non-esterified fatty acids (**NEFA**), total protein and triglycerides (**TG**), and the activities of alanine transaminase (**ALT**) and aspartate transaminase (**AST**) were measured [13]. The coefficient of variation (**CV**) within and between assays for these measurements were < 2.9% and < 8%, respectively. Fructose and *myo*-inositol (henceforth referred to as inositol) were analysed as previously reported [21], and the concentrations of IgA, IgG, and IgM were measured in duplicate with a porcine specific ELISA kit (Bethyl Laboratories Inc., Montgomery, TX) according to the manufacturer’s instructions, with a within and between CV of 2.3% and 4.4%, respectively. The detection range for each Ig was; IgA: 7.8–500, IgG: 15.6–1000 and IgM: 15.6–1000 (ng / mL). Insulin-like growth factor 1 (**IGF1**) was measured by ELISA (Mediagnost, Reutlingen, Germany: Product No: E20), and the IGF binding proteins 2, 3, 4 and 5 (**IGFBP-2–5**) were measured via Western ligand blot, as previously described [22]. For IGF1, the CV within and between assays for these measurements were < 1.3% and < 2.6%, respectively.

### Stomach content and duodenum and liver tissue amino acid concentrations

Plasma samples collected at birth, 4 h after birth and at slaughter (5 d), as well as gastric contents, duodenal mucosa and liver tissue were analysed for the concentration of free proteinogenic amino acids (**FAA**) and amino acid metabolites (i.e., 1-, 3-methylhistidine, alpha-aminoadipic acid, α-aminoisobutyric acid, beta-alanine, citrulline, gamma-aminobutyric acid, hydroxyproline (**HyP**), ornithine and taurine, as well as the peptides anserine and carnosine) as previously described [23]. In addition, the concentrations of protein-bound amino acids (**PBAA**) in the duodenal and liver tissue were determined as well. Due to the high concentrations of Gln and Ala in the stomach content, samples had to be diluted four-fold to enable quantification of Ala and Gln. As a consequence of the dilution, the concentrations of cysteine, methionine and proline were too low to be quantified, with the quantification limit per g of dry matter as follows: cysteine (8 µmol), methionine (2 µmol) and proline (40 µmol).

### Duodenum and liver biochemical indices and duodenum, colon and liver fractional protein synthesis rate

Liver total DNA, RNA and protein, and duodenal mucosa RNA and duodenal tissue protein were extracted, and the biochemical indices (**BCI**) were calculated as previously described [23]. Duodenal mucosa DNA could not be extracted due to the small amount of available material. Fractional protein synthesis rate (**FPSR**) in duodenal, colon and liver tissue were determined as described [23].

### Duodenum mucosa and liver tissue transcript abundance

Duodenal mucosa and liver tissue RNA (30 μg) extracted for the calculation of BCI was purified using RNeasy minikits (Qiagen, Hilden, Germany) and quantified using a Nanophotometer (Implen GmbH, Munich, Germany). The RNA quality was assessed using a Bioanalyzer 2100 and RNA 6000 Nano kit (Agilent Technologies, Waldbronn, Germany), with an RNA integrity number range of (6–9 (mean 8.04 ± 0.7, duodenum mucosa); 6–8.7 (mean 7.84 ± 0.55, liver tissue)). Purified RNA (500 ng) was reverse transcribed to make cDNA using the SensiFAST™ cDNA Synthesis Kit (Bioline, Berlin, Germany) according to the manufacturer’s instructions. Primers were made by Integrated DNA Technologies (IDT, Antwerp, Belgium), and selected from previous studies or designed using the IDT RealTime qPCR Assay design tool (S2 and S3 Tables). Quantitative real time PCR was performed, and PCR efficiency and quantification cycles calculated as previously reported [23]. The GeNorm applet from qBASEplus selected three reference genes (Heat shock 90kD protein 1, beta (***HSPCB***), Peptidylprolyl isomerase A (***PPIA***), Proteasome 26S Subunit, ATPase 3 (***PSMC3***) from eight candidates (S2 Table), as the most stably expressed across the BiW and Suppl experimental groups used in this study. Reference genes were used to normalize target gene mRNA abundance in the qBASEplus software and the Cq-values were converted into log transformed calibrated normalized relative quantities (Log-CNRQ) values, taking into account amplification efficiencies, inter-run variations, and normalization factors. All data was reported as per the Minimum Information for Publication of Quantitative Real-Time PCR Experiments (MIQE) guidelines [24].

### Plasma metabolome

Targeted metabolite analysis was conducted using a subset of plasma samples (n = 6/group; LBiW-Gln, LBiW-Ala, NBiW-Gln, NBiW-Ala) collected at 5 d, which were selected based on piglet ADG (0–5 d) and liver triglyceride concentrations (5 d) reported in a companion study [10]. The plasma metabolites were measured at the Institute of Clinical Chemistry and Laboratory Medicine of the University Medicine Greifswald. The assay utilized 10 µL plasma and the samples were processed according to the manufacturer’s instructions. Analysis was based on Liquid Chromatography-Electrospray Ionization-Tandem Mass Spectrometry and Flow Injection-Electrospray Ionization-tandem Mass Spectrometry (**LC-MS/MS**) measurements using the MxP® Quant 500 kit (Biocrates Life Sciences AG, Innsbruck, Austria). The method of the MxP® Quant 500 kit conforms to the European Medicines Agency guideline ‘M10 on Bioanalytical Method Validation—Scientific Guidelines’ [25], which implies proof of reproducibility within a given error range. Measurements were performed as described by the manufacturer, with metabolite identification and quantification performed using plate-integrated internal standards (isotope labelled). The limit of detection was set to three times the value of the zero samples (Phosphate buffered saline). The MxP® Quant 500 kit assay procedures, metabolite nomenclature, sample handling and mass spectrometric analyses have been all previously described in detail [26]. After the measurements, the data were uploaded to the Biocrates MetIDQ software, where in a first pre-processing step, the peak integration and the calibration curves were checked before the automatic calculation of the metabolite concentrations was performed. To account for day-to-day variation in performance of the LC-MS/MS platform, up to three quality control samples were added to each plate. The corresponding metabolite concentrations were used for normalization. For each plate the measured concentrations of the metabolites were divided by the median concentration of the quality control samples. Subsequently, for each metabolite the median of the plate-specific medians of the pooled samples was calculated to reset the concentration to the original scale (µM concentrations). For each metabolite the plate-specific coefficient of variation was calculated based on the quality control samples. Only metabolites with a mean coefficient of variation < 30% and at least 20 measurements above the limit of detection were included in the final data set resulting in 424 metabolites used for subsequent analysis. From these 424 metabolites: 20 AA + total AA, 11 AA-metabolites, lactate and glucose were removed as results from these parameters have been published in a companion study [10]. Therefore, 390 plasma metabolites remained to be analysed.

### Multi-block integrative analysis

The DIABLO (Data Integration Analysis for Biomarker discovery using Latent variable approaches) model, implemented in the R package mixOmics [27] was applied to integrate and explore interrelationships among four datasets (“**blocks**”) within the liver tissue: FAA (block 1, *n* = 20), AA-metabolites (block 2, *n* = 12), PBAA (block 3, *n* = 20) and mRNA (block 4, *n* = 29; S4 Table) and duodenum mucosa: FAA (block 1, *n* = 20), AA-metabolites (block 2, *n* = 10), PBAA (block 3, *n* = 20) and mRNA (block 4, *n* = 26; S4 Table). The plasma metabolome data was excluded from the DIABLO analysis as only a subset of animals (*n* = 24 from 48) was assessed, whilst the stomach digesta FAA were not included as only 27 animals had digesta samples.

The DIABLO model was used to identify which variables best discriminated between the following experimental group comparisons (LBiW-Ala vs. LBiW-Gln; NBiW-Ala vs. NBiW-Gln; LBiW-Ala vs. NBiW-Ala and LBiW-Gln vs. NBiW-Gln). Data pre-processing included log-transforming and scaling all data to unit variance before analysis. A design matrix value of 0.1 and three components were used to fit the model, and performance was assessed using 5-fold cross-validation with 10 repeats and balanced error rate. The final model, composed of the number of components based on the lowest balanced error rate using centroid distance, was used to select the optimal number of features for each component and each data block. The main outputs were individual sample plots visualizing the projection of each sample into the DIABLO framework and biplots showing the contribution (loading weights ≥ 0.4) of each selected variable.

### Statistical analysis

Sample size (*n*) was calculated as a 2-level factor combination of (1) BiW (LBiW, NBiW) and (2) supplementation (Ala, Gln), using CADEMO for Windows ANOV-Version 4.03 (2000; BioMath GmbH, Rostock). Alpha, or “the significance cut off” was set at 0.05 and beta, or “failure to reject a false null hypothesis” was set at 0.20. The primary outcome measure used to determine *n* was BW gain. The use of BW gain to determine sample size, may have underpowered the transcriptomic, metabolomic and multivariate analyses, and this should be considered when interpreting the results.

The normality of residuals was tested using the Shapiro–Wilks’s test and Q-Q Plot assessment in SAS (version 9.4; SAS Institute Incorporated). Outliers were defined as 1.5 times the interquartile range greater than the third quartile or 1.5 times the interquartile range less than the first quartile, and once identified, removed if they contributed to data not being normal. Model selection was based on Akaike’s information criterion. Model 1, used for the evaluation of BW, BW gain, plasma FAA profiles, clinical parameters, IGF system and immunoglobulins (IgA, IgG and IgM) at 4 h after birth, was the GLIMMIX procedure of SAS, using a Gaussian model (model statement: distribution = Gaussian, link = Identity), and the fixed effect of BiW (LBiW, NBiW), and Sow as a random effect. Model 2, used for the evaluation of log-transformed stomach content and duodenal and liver tissue FAA and AA-metabolites, duodenal and liver tissue PBAA, duodenal, colon and liver tissue FPSR, duodenal mucosa and liver tissue mRNA abundance, as well as duodenal and liver BCI was the same as Model 1, but included the fixed effect of Suppl (Ala, Gln), and the interaction BiW × Suppl. The SLICE statement was used for performing partitioned analyses of the least-squares means for the BiW × Suppl interaction. Least squares means ± standard errors are reported. Differences were considered significant if Tukey-Kramer test was *P* ≤ 0.05.

Targeted plasma metabolite data were analysed with MetaboAnalyst 6.0 [28]. Data was filtered as follows: metabolites were removed from the dataset if they had a zero value in 20% or more of the samples in each of the experimental groups, samples with a relative standard deviation > 20% and > 20% of near-constant variables using interquartile range. After filtering 338 metabolites remained for analysis, and missing observations were replaced with half of the minimum value of the specific metabolite in the original data. Data were log-transformed and autoscaled. Experimental group pairings (Suppl: LBiW-Gln vs. LBiW-Ala, NBiW-Gln vs. NBiW-Ala; BiW: LBiW-Gln vs. NBiW-Gln, LBiW-Ala vs. NBiW-Ala) were first analysed by principal component analysis to lower data dimensionality and to acquire an overview by presenting trends, groupings, and classify potential outliers. Samples were considered as outliers when they were situated outside the 95% confidence ellipse region of the model. Next, two sample t-tests with unequal group variance with false discovery rates (q-value) correction and a *P*-value threshold of 0.3 were performed on the experimental group pairings to select potentially important metabolites for inclusion in the supervised orthogonal partial least squares discriminate analyses (OPLS-DA). No differences were identified; therefore, the entire metabolomics dataset was included in the subsequent OPLS-DA analyses. This method is a common tool to perform classification and regression in metabolomics by using multivariate regression techniques to optimize separation between different groups [29]. Method-validity was verified with Q2 and R2 permutation testing and after inspection of three important model characteristics (R2X, R2Y and Q2Y). Permutation testing explains the total sum of variation in Y. R2X and R2Y are both goodness-of-fit parameters and Q2Y is a goodness-of-prediction parameter. For these three model characteristics, a parameter value > 0.5 indicates good model quality.

## Results

### Plasma free amino acid profile, clinical parameters, immunoglobulins, IGF1 and IGF binding proteins at 4 h after birth

At birth and 4 h after birth, LBiW piglets were lighter than NBiW (*P <* 0.001; Table 1). At 4 h after birth the plasma AA cysteine concentration (*P =* 0.03) was lower in LBiW than in NBiW littermates (*P =* 0.003; Table 1). Concentrations of albumin, bilirubin (*P <* 0.001), glucose (*P =* 0.04) and total protein (*P <* 0.001) were lower, whilst ALT (*P =* 0.006), inositol (*P =* 0.001) and lactate (*P =* 0.007) were higher in plasma of LBiW compared with NBiW littermates (Table 1). The plasma concentrations of IGF1 (*P <* 0.001), IGFBP-3 (*P =* 0.003) and IGFBP-4 (*P =* 0.005) as well as IgA, IgG (*P <* 0.001) and IgM (*P =* 0.01) were lower in LBiW compared to NBiW littermates (Table 1). The results of parameters with no significant differences are presented in S5 Table.

**Table 1:** Birth weight and body weight 4 h after birth, as well as concentrations of clinical-chemistry and amino acid parameters in the plasma of male low (LBiW) and normal (NBiW) birthweight piglets 4 h after birth.

Item^1^	LBiW	NBiW	*P-*values
Bodyweight (kg)			
Birth	1.10 ± 0.02	1.50 ± 0.02	< 0.001
4 h after birth	1.12 ± 0.02	1.52 ± 0.03	< 0.001
Amino acids (µmol/L)			
Cysteine	108.78 ± 6.34	130.85 ± 6.56	0.027
Clinical Parameters			
Alanine transaminase (U/L)	32.60 ± 2.17	25.40 ± 2.35	0.006
Albumin (g/L)	6.96 ± 0.24	8.26 ± 0.27	< 0.001
Bilirubin (µmol/L)	4.34 ± 0.45	6.54 ± 0.48	< 0.001
Glucose (mmol/L)	4.33 ± 0.31	5.03 ± 0.31	0.041
Inositol (mmol/L)	4.51 ± 0.37	3.22 ± 0.37	0.001
Lactate (mmol/L)	3.64 ± 0.22	3.01 ± 0.22	0.007
Total Protein (g/L)	26.73 ± 1.27	31.12 ± 1.27	< 0.001
Insulin-Like Growth Factor (IGF) system (ng/mL)^2^			
IGF1	39.30 ± 2.61	56.57 ± 2.68	< 0.001
IGF Binding Protein 3	416.80 ± 32.24	576.80 ± 33.52	0.003
IGF Binding Protein 4	112.55 ± 6.50	132.68 ± 6.50	0.005
Immunoglobulins (Ig, mg/mL)			
IgA	1.40 ± 0.37	2.39 ± 0.37	< 0.001
IgG	3.01 ± 0.65	5.14 ± 0.65	< 0.001
IgM	0.47 ± 0.11	0.71 ± 0.11	0.014

^1^Values are LSM ± SEM; *n* = 48, *n* = 12 / subgroup.

^2^*n* = 24–25, *n* = 14–15 LBiW, *n* = 10 NBiW.

### Free amino acid profiles of stomach contents

Concentrations of Ala, Gln (*P* < 0.001) and glutamate (**Glu**; *P* = 0.005) in stomach contents were affected by Suppl (S6 Table). The concentration of Ala was higher in Ala-supplemented (*P* < 0.001) littermates compared to those supplemented with Gln, whilst Gln (*P* < 0.001) and Glu concentrations (LBiW-Gln vs. LBiW-Ala: *P* = 0.02, NBiW-Gln vs. NBiW-Ala: *P* = 0.03) were higher in Gln compared to Ala-supplemented littermates (S6 Table).

### Amino acid concentrations in duodenal and liver tissue and mRNA abundance in the duodenal mucosa and the liver tissue

To investigate the interrelationships between the duodenal FAA, AA-metabolites, PBAA and mRNA data blocks within BiW or Suppl groups, an analysis was conducted using DIABLO. When the LBiW-Ala and LBiW-Gln groups were analysed, the model achieved a balanced error rate of 0.23, and the cross-validated area under the curve (0.97, *P* = 0.015) indicating the model’s robust discriminative performance. Subsequent results showed moderate-to-strong correlations between the duodenal FAA and AA-metabolites blocks (r = 0.58) and between FAA and mRNA blocks (r = 0.64), with significant group separation observed with these pairings (S1A Fig). Individual projections across blocks, showed that the FAA block was primarily driving the separation between the LBiW-Ala and LBiW-Gln groups (Fig 1A). A DIABLO biplot showed clear discrimination between the LBiW-Ala and LBiW-Gln groups along Component 1, indicating strong separation (Fig 1B). The separation appeared to be driven by the FAA Gln and Ala, the AA-metabolites Car and citrulline (**Cit**), and the mRNA transcripts *TJP1, SLC7A8, GSS and GSR* (Fig 1B). A univariate analysis (S7, S8 and S9 Tables) confirmed that these parameters were significantly different between LBiW-Gln and LBiW-Ala piglets (Fig 2A-H).

**Fig 1:**
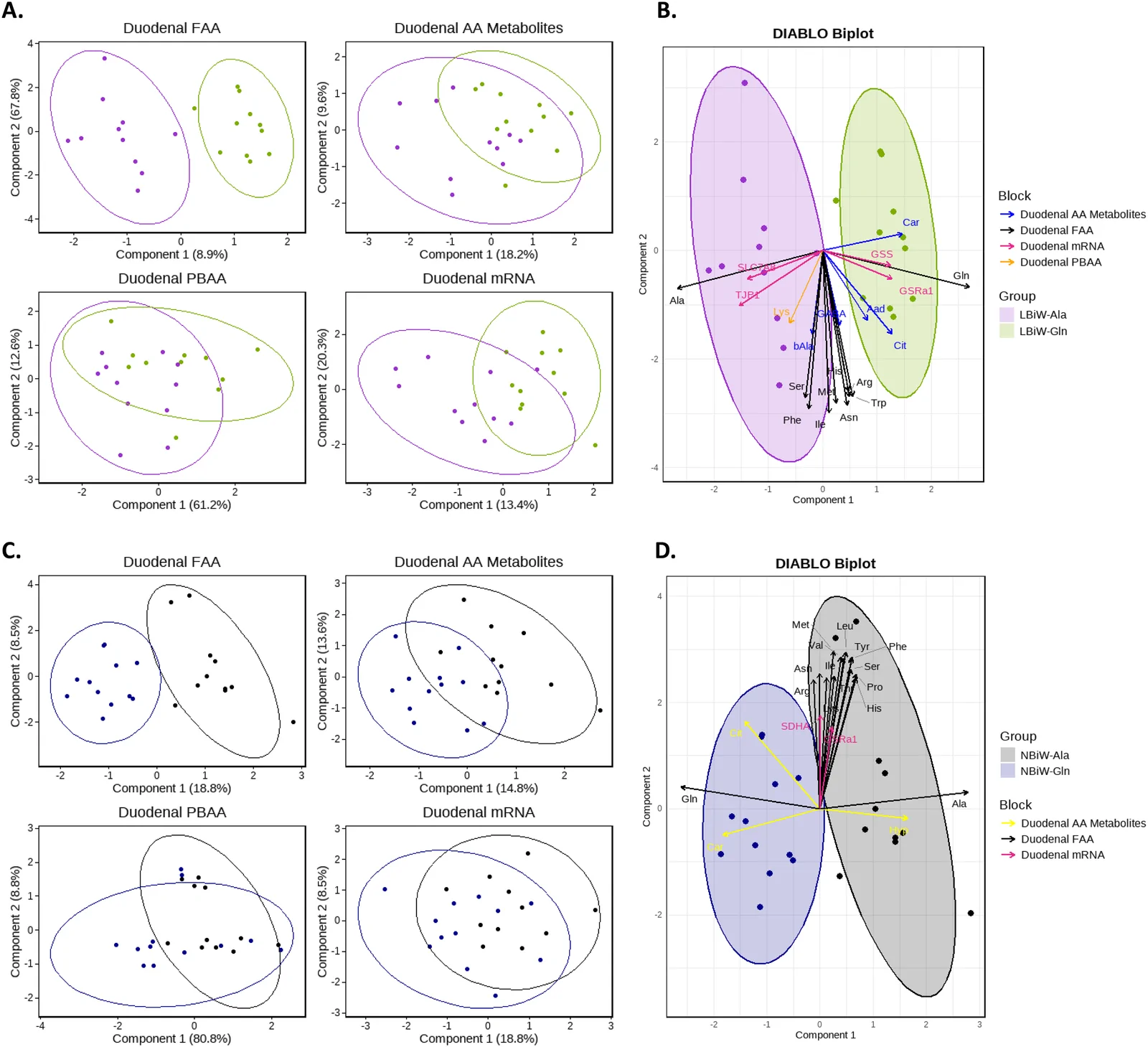
Data Integration Analysis for Biomarker discovery using Latent variable approaches for Omics studies (DIABLO) analysis of the four duodenal datasets. DIABLO plots of all 76 duodenal variables assigned to four datasets (‘blocks’) and measured in 48 samples. Shown are the sample plots for all four blocks: ‘Free amino acids’, ‘AA-metabolites’, ‘protein-bound amino acids and ‘mRNA’ for the (**A**) LBiW-Ala vs. LBiW-Gln and (**C**) NBiW-Ala vs. NBiW-Gln comparisons, and biplots of which variables from these blocks contribute to the separation between the (**B**) LBiW-Ala vs. LBiW-Gln and (**D**) NBiW-Ala vs. NBiW-Gln comparisons.

**Fig 2:**
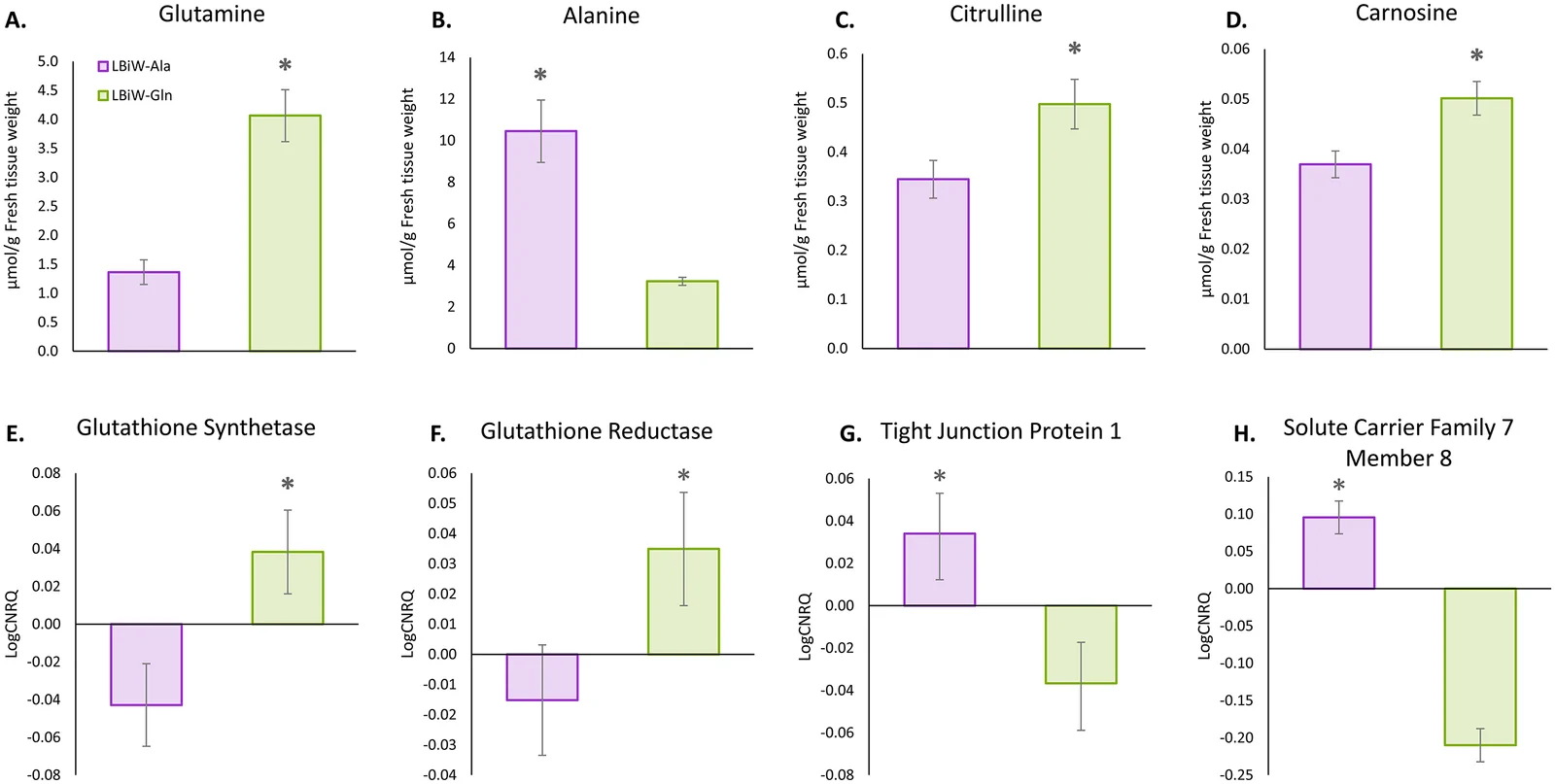
Bar graphs of duodenal variables that contribute to separation of the LBiW-Gln and LBiW-Ala experimental groups in the DIABLO analysis. Shown are the bar graphs for the ‘blocks’: ‘Free amino acids’ (**A: Glutamine, B: Alanine**), ‘AA-metabolites’ (**C: Citrulline, D: Carnosine**) and ‘mRNA’ (**E: Glutathione Synthetase, F: Glutathione Reductase, G: Tight Junction Protein 1, H: Solute Carrier Family 7 Member 8**) for the LBiW-Gln vs. LBiW-Ala comparison. A significant difference identified using the GLMMIX procedure in SAS is shown by an asterisk (*P* < 0.05).

When the NBiW-Ala and NBiW-Gln groups were analysed, the model achieved a balanced error rate of 0.19, and the cross-validated area under the curve (0.95, *P* = 0.033) indicating the model’s robust discriminative performance. Results showed moderate-to-strong correlations only between the FAA and AA-metabolites blocks (r = 0.77), with weak group separation (S1B Fig). Individual projections across blocks, showed that the duodenal FAA block was primarily driving the separation between the NBiW-Ala and NBiW-Gln groups (Fig 1C). A DIABLO biplot showed clear discrimination between the NBiW-Ala and NBiW-Gln groups along Component 1, indicating strong separation (Fig 1D), with separation appearing to be driven by the FAA Gln and Ala and AA-metabolites Cit, Car and HyP (Fig 1D). A univariate analysis (S7 Table) confirmed that, with the exception of HyP, these parameters, differed significantly between NBiW-Gln and NBiW-Ala piglets (Fig 3A-E). None of the models achieved a balanced error rate below 0.5 for the parameters in liver tissue, which indicates poor classification performance; therefore, no further analysis of this dataset was performed.

**Fig 3:**
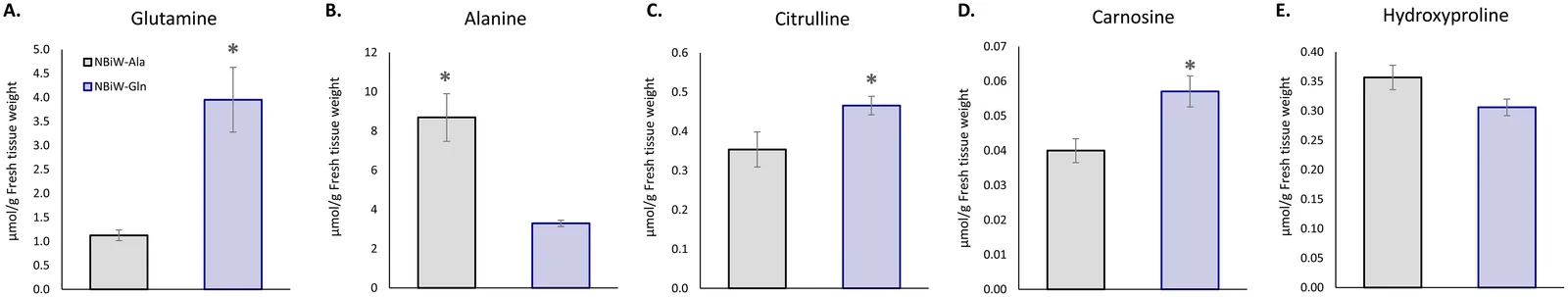
Bar graphs of duodenal variables that contribute to the separation of the NBiW-Gln and NBiW-Ala experimental groups in the DIABLO analysis. Shown are the bar graphs for the ‘blocks’: ‘Free amino acids’ (**A: Glutamine, B: Alanine**) and ‘AA-metabolites’ (**C: Citrulline, D: Carnosine, E: Hydroxyproline**) for the NBiW-Ala vs. NBiW-Gln comparisons. A significant difference identified using the GLMMIX procedure in SAS is shown by an asterisk (*P* < 0.05).

### Biochemical indices of duodenum and liver and fractional protein synthesis rate in the duodenum, colon and the liver

The duodenum protein (*P* = 0.05) and RNA (*P* = 0.01) concentrations were affected by Suppl. The concentration of duodenum mucosa RNA was higher in LBiW-Ala than LBiW-Gln (*P* = 0.02: S10 Table). The liver ratio of protein to RNA (*P* = 0.03) was affected by BiW. The concentration of protein in the liver was higher in LBiW-Gln than LBiW-Ala (*P* = 0.05), whilst the ratio of protein to RNA was higher and the ratio of RNA to DNA was lower (*P* = 0.05) in LBiW-Ala than NBiW-Ala littermates (S10 Table). Colon FSPR was higher in NBiW-Ala compared to LBiW-Ala (*P* = 0.04), whilst no difference in liver or duodenum FPSR was observed (S10 Table).

### Plasma metabolome

Any potential discrimination between the four experimental groups (LBiW-Gln, LBiW-Ala, NBiW-Gln, NBiW-Ala) was clarified by OPLS-DA, in multiple two-way (LBiW-Gln vs. LBiW-Ala, LBiW-Gln vs. NBiW-Gln, LBiW-Ala vs. NBiW-Ala and NBiW-Gln vs. NBiW-Ala) experimental group comparisons. Despite the good separation achieved between the experimental groups, and high total sum of variation explained by the model (R2Y: LBiW-Gln vs. LBiW-Ala = 0.999, LBiW-Gln vs. NBiW-Gln = 0.998, LBiW-Ala vs. NBiW-Ala = 0.996 and NBiW-Gln vs. NBiW-Ala  =  0.864), the estimation of model predictive performance was modest (Q2: LBiW-Gln vs. LBiW-Ala = 0.340, LBiW-Gln vs. NBiW-Gln = 0.239, LBiW-Ala vs. NBiW-Ala = 0.298 and NBiW-Gln vs. NBiW-Ala  =  0.124). The OPLS-DA model was then validated using permutation testing with *P*-values for permutation testing as follows: (pR2Y: LBiW-Gln vs. LBiW-Ala = 0.439, LBiW-Gln vs. NBiW-Gln = 0.911, LBiW-Ala vs. NBiW-Ala = 0.265 and NBiW-Gln vs. NBiW-Ala  =  0.502) and (pQ2: LBiW-Gln vs. LBiW-Ala = 0.352, LBiW-Gln vs. NBiW-Gln = 0.577, LBiW-Ala vs. NBiW-Ala = 0.317 and NBiW-Gln vs. NBiW-Ala  =  0.285), thus showing no statistically significant validation of the separation achieved between groups. In addition, results from the two-sample t-tests and ANOVA showed that there were no significantly different metabolites between the experimental groups.

## Discussion

We have previously reported that in 5 d Gln and Ala-supplemented piglets the plasma concentration of their respective supplemented AA were significantly increased [10]. However, only NBiW piglets supplemented with Gln had higher concentrations of jejunal Gln compared to Ala-supplemented NBiW piglets, whilst no difference in jejunal Ala concentrations was observed [23]. It was also observed that Ala-supplemented LBiW piglets had higher plasma triglycerides than Gln-supplemented LBiW piglets, and higher liver TG than NBiW supplemented with Ala [10].

Therefore, we hypothesized that Gln and Ala absorption occurred at a more proximal section of the small intestine, that liver AA and lipid metabolism had been altered in response to Gln supplementation, and that these changes would be associated with alterations in the plasma metabolome, and together, may help provide information on how Gln improves LBiW piglet BW compared to LBiW piglets supplemented with Ala, as observed in a companion study [10].

### Low birthweight piglet selection

At birth and 4 h after birth, the LBiW piglets selected for the current study were lighter than their NBiW littermates and it has been reported that there is a positive relationship between foetal weight and albumin levels [30]. This relationship may explain why plasma albumin concentrations at 4 h after birth was lower in LBiW than NBiW piglets from this study. A function of albumin is to act as a carrier of bilirubin, which was lower in LBiW than NBiW piglets. Bilirubin is a breakdown product of red blood cells, and in neonatal LBiW piglets the concentration of red blood cells has been shown to be lower than in NBiW piglets [31], an indication that LBiW piglets in this study may have had a lower red blood cell concentration than NBiW piglets.

It has been reported that LBiW piglets can take longer to make their first suckle and / or their colostrum intake in the first 24 h of life is lower than their NBiW littermates [32]. In a companion study, we found no difference in the time to first suckle, but did observe a trend towards lower colostrum intake in the first 24 h among the LBiW piglets used in this study [10]. Four h after birth, both immunoglobulins and glucose in new born piglets derive exclusively or predominantly from colostrum; their concentrations in plasma were lower in LBiW than NBiW piglets, suggesting lower colostrum intake. Together, the albumin and immunoglobulin result explains why total protein, which consists primarily of immunoglobulins and albumin [33], was lower in LBiW than NBiW pigs. Lower colostrum intake has also been shown to negatively impact circulating IGF1 [34], which were lower in LBiW compared to NBiW littermates in this study. Over 99% of IGF1 (and IGF2) is bound to binding proteins, with almost 75% bound to IGFBP-3, the serum concentration of which correlates with that of IGF1 [35]. We therefore suspect that the lower IGFBP-3 levels in LBiW piglets are a consequence of the lower circulating IGF1 concentrations. As for IGFBP-4, it has an inhibitory role on IGF1 function, and its concentration is independent of IGF1 [35]. It should be noted, that the current IGF1, IGFBP-3 and IGFBP-4 results contradict those of a previous study, conducted with another cohort of piglets from the same facility of FBN [13]. In this earlier study, we observed no difference in plasma IGF1 concentrations between LBiW and NBiW piglets 4 h after birth, a finding that cannot be explained by the information currently available.

In the current study, we also observed higher plasma concentrations of inositol in LBiW compared to NBiW piglets. Higher plasma concentrations of inositol at birth are a biomarker of low birthweight in infants and pigs [36], where inositol is proposed to play a protective or adaptive role in the response to metabolic stress [37]. Why plasma inositol is higher in neonatal LBiW compared to their NBiW piglets is due to carryover from the *in-utero* environment, where we have previously reported higher concentrations in light compared to heavy 94 day of gestation foetuses [38]. However, the mechanisms behind the observed higher plasma concentration of inositol in LBiW compared to NBiW neonatal piglets is currently unknown. Lactate was also observed to be higher in LBiW than NBiW piglets and it has been reported that piglets with higher lactate levels not only have a lower birthweight, but show less vitality and consume less colostrum than those with a lower lactate concentration [39]. Lactate is used as an indicator of potential oxygen deprivation which has been shown in pigs to possibly be a consequence of a longer time to birth or placental insufficiency, which leads to liver damage and increased levels of ALT [40], all of which were observed in the LBiW compared to NBiW piglets in this study. Therefore, taken together, this collection of results shows that the LBiW piglets selected for this study were less mature at birth with a reduced potential to thrive than their NBiW littermates.

### Glutamine supplementation and amino acid profiles of stomach contents

To our knowledge, this is the first study to report AA profiles in the stomach content of suckling piglets supplemented with Gln or Ala. The analysis of the FAA pool in the stomach content 2 h after supplementation showed that the concentration of Gln and Ala of supplemented piglets was still so high that all content samples had to be diluted four-fold to enable quantification. In a pretrial, the plasma concentration of Gln at 2 h after oral administration of a glutamine bolus was 80% of the peak value (1.06 mmol/l) which occurred at 45 min [10], suggesting that whilst Gln concentrations in stomach content are high, they may not represent the maximum concentration to be observed during our study. This indicates that not all supplemental glutamine passed through the stomach within 2 h. In addition to higher Gln concentrations, we also observed higher concentrations of Glu in the stomach content of Gln-supplemented compared to Ala-supplemented piglets, which is likely due to the acidic environment of the stomach accelerating hydrolysis of the Gln to Glu.

### Glutamine supplementation and duodenal amino acid profiles and mRNA abundance

The results of the DIABLO analysis showed that some of the key duodenal tissue parameters driving the separation between the supplementation groups, regardless of BiW were Gln, Car and Cit for the Gln-supplemented piglets and Ala for the Ala-supplemented piglets. Subsequent mixed-model analysis showed that the duodenal concentration of Gln, Car and Cit were higher in Gln-supplemented piglets, and Ala in Ala-supplemented piglets, a result that contrasts with those from a companion study which reported that the concentration of Gln, Car, Cit, Ala was not different in the mid-jejunum tissue and digesta of the same piglets reported in this study [23]. Thus, taken together, these results show that the majority of the absorption, and potential metabolism of the supplemental AA may occur at the duodenum. The increased absorption and / or metabolism through the TCA cycle, and subsequent generation of H + may explain the higher duodenal tissue Car concentrations in Gln-supplemented piglets, processes which can lead to lower intracellular pH [41]. Under these conditions Car has been reported to act as an intracellular buffer to maintain intracellular pH levels [42]. The higher duodenal tissue Cit concentration of Gln-supplemented piglets suggests metabolism of supplemental Gln into Cit [43]. We have previously reported that no difference in plasma Cit concentration was observed in piglets used in this study; however, the piglets in the in NBiW-Gln group had significantly higher plasma concentrations of Arg compared to those in the NBiW-Ala group, whereas for LBiW-Gln compared to LBiW-Ala piglets this was only a trend [10], indicating potential metabolic differences between the duodenum of LBiW and NBiW piglets when orally supplemented with 1 g / kg BW Gln. Plasma Arg is a picture of whole body Arg synthesis and metabolism, and the intestine and kidney have been shown to synthesize Arg from Cit [43], but as there is no difference in duodenal concentrations of Cit between LBiW and NBiW piglets supplemented with Gln, it is possible that more Arg is produced in the kidney of NBiW than LBiW, or that NBiW piglets consume more milk and thus Arg. Either way, why these scenarios may occur in NBiW piglets supplemented with Gln and not LBiW piglets remains to be elucidated.

These differences may help to explain why the mRNA abundance of duodenal mucosa glutathione reductase and glutathione synthetase were higher only in LBiW-Gln compared to LBiW-Ala piglets. The glutathione reductase mRNA encodes a protein that reduces oxidized glutathione back to reduced glutathione (GSH), whilst glutathione synthetase mRNA encodes a protein that synthesizes additional reduced GSH [44]. Reduced glutathione is an antioxidant that helps to protect tissues against free-radicals and remove toxins [44], and these results suggest that the duodenum of LBiW-Gln piglets may be preparing to produce more reduced GSH, or is actively doing so in a compensatory or protective response to increased cellular stress. Why this change was observed in the duodenum of LBiW and not NBiW piglets may lie in the negative effect a low birthweight has on small intestine development, especially the antioxidative system, which is impaired in LBiW piglets [45,46]. Increased Gln transport and metabolism by the duodenum of LBiW piglets may have increased oxidative stress, that required some of the supplemental Gln to be diverted to the production of GSH. This may also explain the lower abundance of SLC7A8 in LBiW-Gln compared to LBiW-Ala and not NBiW-Gln compared to NBiW-Ala. The SLC7A8 AA transporter maintains intracellular AA pools by mediating the influx of extracellular AA in exchange with the efflux of highly concentrated intracellular AA, and is found on the apical side of the enterocyte [47]. Therefore, the downregulation could lower export of Gln into the blood stream, and spare it for processes such as GSH synthesis. Increased duodenal oxidative stress could also be associated with the lower duodenal abundance of tight junction protein 1 mRNA in LBiW-Gln piglets compared to LBiW-Ala piglets. If the tissue is exposed to significant oxidative stress, this stress could impair the intestinal barrier, as has been reported in infections with enterotoxigenic *Escherichia coli* [48] and during weaning [49], leading to a downregulation of tight junction protein 1 mRNA expression. Further, oxidative stress has been associated with destruction of a cell’s ribosomes [50] and this could help to explain the reduced total RNA levels in the duodenal tissue, 80–85% of which consists of ribosomal RNA, in LBiW-Gln piglets compared to LBiW-Ala piglets. However, this does appear to have impacted protein synthesis, as assessed by no difference in duodenal total protein and FPSR between LBiW-Gln and LBiW-Ala piglets, suggesting a compensatory mechanism to maintain protein synthesis.

### Glutamine supplementation and liver amino acid profiles and biochemical indices

We have previously observed that the liver of the LBiW-Ala piglets used in this study had higher TAG levels than NBiW-Ala, and we observed higher plasma concentrations of Gln, Car and Arg in Gln-supplemented pigs and Ala in Ala-supplemented pigs [10]. As the liver is the central organ for fat metabolism and AA partitioning, we hypothesized that we would observe changes in liver FAA profiles and genes associated with AA transport and metabolism and lipid metabolism that distinguish Gln from Ala-supplemented piglets. Results from the DIABLO analysis showed that there was no significant separation of the experimental group comparisons when the liver parameters of FAA, PBAA and mRNA abundance were analysed. Whilst no comparable studies have yet been conducted on hepatic metabolism in Gln supplemented suckling piglets, there is one study that was carried out on weaned piglets [51]. In weaned piglets fed a Gln-supplemented diet for 30 d, a possible downregulation of hepatic glycolysis, and changes in fructose metabolism were observed [51]. Downregulation of glycolysis has also been observed in jejunal enterocytes isolated from 0 to 21 d piglets and incubated with Gln [52], and taken together results from these two studies suggest changes in hepatic metabolism may have occurred that were not assessed in this study. The biochemical indices parameter of total protein was higher in LBiW-Gln than NBiW-Gln, but as there was no difference in total PBAA between the two groups, this result remains difficult to interpret, as does the higher protein synthetic efficiency in LBiW-Ala compared to NBiW-Ala piglets.

### Glutamine supplementation and the plasma metabolome

This is the first study to report the plasma metabolome of suckling piglets supplemented with Gln, and from the 338 plasma metabolites analysed no changes compared to piglets supplemented with Ala were observed. Whilst no other studies investigating the plasma metabolome in suckling piglets supplemented with Gln have been conducted, two studies have been conducted in female pigs weaned at 21 d [51,53]. They analysed the serum metabolome after 7 [53] and 30 d [51] of Gln supplementation, and identified 5 and 12 metabolites respectively, that were different between Gln and Ala-supplemented pigs. Of the metabolites identified in these two studies only one in the 7 d samples, creatinine, and two in the 30 d samples, proline and tyrosine, were assessed in the targeted metabolomics kit used in this study, with proline and tyrosine being removed from the final analysis as they were reported previously [10]. Thus, taken together, these results suggest that Gln supplementation in pigs may have a very minimal impact on the circulating metabolome. We acknowledge that the physiological state of suckling and weaning pigs are different, and there may be metabolites not assessed in either study that are different between Gln and Ala-supplemented pigs. In addition, 6 animals per experimental group may not have been enough to detect differences in the targeted metabolites measured in this study.

### Glutamine supplementation and colon fractional protein synthesis rate

Lower colon FPSR was observed in LBiW-Ala compared to NBiW-Ala, yet no other differences between the experimental groups was observed. This is in contrast to a previous study where we reported FPSR in the jejunum [23] and in the current study where we investigate duodenal and liver FPSR, and no differences between any of the experimental groups was observed. Protein synthesis in the colon has been associated with changes in the concentration of short-chain fatty acids (SCFA) [54] and/or biogenic amines (BA) [55] in the colon digesta. In a companion study we reported that the concentration of SCFA and BA, and the abundance of microbiota associated with SCFA and BA production in the colon digesta of the piglets reported in this study did not differ between the experimental groups [11]. Therefore, it is currently unknown why colon FPSR was lower in LBiW-Ala compared to NBiW-Ala.

## Conclusions

This study showed that 2 h after oral supplementation with Gln there were still significantly high levels of free Gln in the stomach content and duodenal tissue of suckling piglets. From the stomach content results we conclude that not all the supplemental Gln had passed into the small intestine within the observational period of 2 h and provides a potential explanation as to why the reported plasma free Gln concentrations in a pre-trial using the same pigs are still 80% of the peak which was reached 45 min after bolus administration. In the duodenum tissue of LBiW and NBiW piglets supplemented with Gln, higher Cit concentrations compared to piglet supplemented with Ala, may be a shared metabolism of Gln to Cit, potentially explaining previously published results of higher plasma Arg. Higher duodenal Car in Gln supplemented piglets may be an attempt to regulate intracellular pH, a potential result of increased transport and / or metabolism of additional Gln. Finally, we observed higher mRNA abundance of genes associated with GSH synthesis in the duodenum of LBiW piglets supplemented with Gln, which may be a response to increased cellular stress. The hypothesized changes in duodenal intracellular pH or GSH synthesis remain speculative and further *in vivo* studies need to be conducted to validate these conclusions. Additionally, studies using stable-isotope labelled Gln together with an unlabelled Gln bolus should be conducted to better understand the *in vivo* metabolic fate of the supplemental Gln in the duodenum of suckling piglets.

## Supporting information

S1 Fig Diagnostic plot from DIABLO multiblock sparse partial least square discriminant analysis of duodenal (A) and liver (B) datasets from low (LBiW) and normal (NBiW) birthweight (BiW) piglets supplemented with glutamine or alanine from 24 hours after birth to 5 days of age, showing the correlation between components from each dataset. (DOCX)

S1 Table Characteristics of experimental litters selected for the study. (DOCX)

S2 Table Real time PCR primer sequences and product size for reference mRNA in duodenal mucosa and liver tissue. (DOCX)

S3 Table Real time PCR primer sequences and product size for target mRNA in duodenal mucosa and liver tissue. (DOCX)

S4 Table List of variables included in the DIABLO analysis. (DOCX)

S5 Table Bodyweight gain, and concentrations of clinical-chemistry and amino acid parameters in plasma of low (LBiW) and normal (NBiW) birthweight piglets at birth (0 h). (DOCX)

S6 Table Stomach content of free amino acid and amino acid (AA) metabolite profiles in low (LBiW) and normal (NBiW) birthweight (BiW) piglets supplemented with glutamine or alanine from 24 h after birth to 5 d of age. (DOCX)

S7 Table Duodenal tissue free amino acid and amino acid (AA) metabolite profiles in low (LBiW) and normal (NBiW) birthweight (BiW) piglets supplemented with glutamine or alanine from 24 h after birth to 5 d of age. (DOCX)

S8 Table Duodenal tissue protein-bound amino acid profiles in low (LBiW) and normal (NBiW) birthweight (BiW) piglets supplemented with glutamine or alanine from 24 h after birth to 5 d of age. (DOCX)

S9 Table Duodenal mRNA abundance in low (LBiW) and normal (NBiW) birthweight (BiW) piglets supplemented with glutamine or alanine from 24 h after birth to 5 d of age. (DOCX)

S10 Table Liver, duodenum and colon biochemical indices and fractional protein synthesis rate (FPSR) in low (LBiW) and normal (NBiW) birthweight (BiW) piglets supplemented with glutamine or alanine from 24 h after birth to 5 d of age. (DOCX)

## References

[1] Harper J, Bunter KL. Review: Improving pig survival with a focus on birthweight: A practical breeding perspective. Animal. 2024;18 Suppl 1:100914. doi: 10.1016/j.animal.2023.100914 37574357

[2] Hu S, Long DW, Bazer FW, Burghardt RC, Johnson GA, Wu G. Glycine supplementation enhances the growth of sow-reared piglets with intrauterine growth restriction. Animals (Basel). 2025;15(13):1855. doi: 10.3390/ani15131855 40646755 PMC12249007

[3] Long DW, Long BD, Nawaratna GI, Wu G. Oral administration of L-arginine improves the growth and survival of sow-reared intrauterine growth-restricted piglets. Animals (Basel). 2025;15(4):550. doi: 10.3390/ani15040550 40003032 PMC11851912

[4] Wessels AG, Simongiovanni A, Zentek J. Impact of dietary supplementation of L-Arginine, L-Glutamine, and the combination of both on nursing performance of multiparous sows. Transl Anim Sci. 2022;7(1):txac169. doi: 10.1093/tas/txac169 36655231 PMC9838099

[5] Ji FJ, Wang LX, Yang HS, Hu A, Yin YL. Review: The roles and functions of glutamine on intestinal health and performance of weaning pigs. Animal. 2019;13(11):2727–35. doi: 10.1017/S1751731119001800 31407650

[6] Chalvon-Demersay T, Luise D, Le Floc’h N, Tesseraud S, Lambert W, Bosi P, et al. Functional amino acids in pigs and chickens: Implication for gut health. Front Vet Sci. 2021;8:663727. doi: 10.3389/fvets.2021.663727 34113671 PMC8185281

[7] Da Silva Fonseca L, Moreira RHR, Palencia JHP, Lanferdini E, Chaves RF, Junior SRS, et al. Effects of maternal L‑glutamine supplementation during late gestation on litter performance. Soyh A J An Sci. 2024;54(1):125–34. doi: 10.4314/sajas.v54i1.14

[8] Zhu Y, Li T, Huang S, Wang W, Dai Z, Feng C, et al. Maternal L-glutamine supplementation during late gestation alleviates intrauterine growth restriction-induced intestinal dysfunction in piglets. Amino Acids. 2018;50(9):1289–99. doi: 10.1007/s00726-018-2608-5 29961143

[9] Yang XF, Qin JF, Wang L, Gao KG, Zheng CT, Huang L, et al. Improved milk glutamine level and growth performance of suckling piglets by glutamine supplementation in maternal diet. Ann Anim Sci. 2018;18(2):441–52. doi: 10.1515/aoas-2017-0040

[10] Li Z, Sciascia M, Görs S, Nguyen N, Rayatdoost Baghal F, Schregel J, et al. Glutamine supplementation moderately affects growth, plasma metabolite and free amino acid patterns in neonatal low birth weight piglets. Br J Nutr. 2022;128(12):2330–40. doi: 10.1017/S0007114522000459 35144703 PMC9723486

[11] Schulze Holthausen J, Schregel J, Sciascia M, Li Z, Tuchscherer A, Vahjen W, et al. Effects of oral glutamine supplementation, birthweight and age on colonic morphology and microbiome development in male suckling piglets. Microorganisms. 2022;10(10). doi: 10.3390/microorganisms10101899 36296176 PMC9612066

[12] Schregel J, Schulze Holthausen J, Sciascia M, Görs S, Li Z, Tuchscherer A, et al. Acute and persistent effects of oral glutamine supplementation on growth, cellular proliferation, and tight junction protein transcript abundance in jejunal tissue of low and normal birthweight pre-weaning piglets. PLoS One. 2024;19(1):e0296427. doi: 10.1371/journal.pone.0296427 38165864 PMC10760696

[13] Sciascia M, Buchallik-Schregel J, Li Z, Görs S, Tuchscherer A, Viergutz T, et al. Association of glutamine supplementation during the early suckling period with growth, weaning, and lipopolysaccharide induced stress in low birthweight piglets. J Anim Sci. 2025;103:skaf296. doi: 10.1093/jas/skaf296 40884114 PMC12448690

[14] Liesegang A. «Empfehlungen zur Energie- und Nährstoffversorgung von Schweinen». Schweizer Archiv für Tierheilkunde. 2008;150(2):84–84. doi: 10.1024/0036-7281.150.2.84b

[15] Baxter EM, Jarvis S, Palarea-Albaladejo J, Edwards SA. The weaker sex? The propensity for male-biased piglet mortality. PLoS One. 2012;7(1):e30318. doi: 10.1371/journal.pone.0030318 22272334 PMC3260262

[16] van den Bosch M, van de Linde IB, Kemp B, van den Brand H. Disentangling litter size and farrowing duration effects on piglet stillbirth, acid-base blood parameters and pre-weaning mortality. Front Vet Sci. 2022;9:836202. doi: 10.3389/fvets.2022.836202 35529832 PMC9071363

[17] Oliviero C, Heinonen M, Valros A, Peltoniemi O. Environmental and sow-related factors affecting the duration of farrowing. Anim Reprod Sci. 2010;119(1–2):85–91. doi: 10.1016/j.anireprosci.2009.12.009 20053511

[18] Haynes TE, Li P, Li X, Shimotori K, Sato H, Flynn NE, et al. L-Glutamine or L-alanyl-L-glutamine prevents oxidant- or endotoxin-induced death of neonatal enterocytes. Amino Acids. 2009;37(1):131–42. doi: 10.1007/s00726-009-0243-x 19189199

[19] Wang X, Wu W, Lin G, Li D, Wu G, Wang J. Temporal proteomic analysis reveals continuous impairment of intestinal development in neonatal piglets with intrauterine growth restriction. J Proteome Res. 2010;9(2):924–35. doi: 10.1021/pr900747d 19938879

[20] Rehfeldt C, Lang IS, Görs S, Hennig U, Kalbe C, Stabenow B, et al. Limited and excess dietary protein during gestation affects growth and compositional traits in gilts and impairs offspring fetal growth. J Anim Sci. 2011;89(2):329–41. doi: 10.2527/jas.2010-2970 20889684

[21] Görs S, Kucia M, Langhammer M, Junghans P, Metges CC. Technical note: Milk composition in mice--methodological aspects and effects of mouse strain and lactation day. J Dairy Sci. 2009;92(2):632–7. doi: 10.3168/jds.2008-1563 19164675

[22] Wirthgen E, Höflich C, Spitschak M, Helmer C, Brand B, Langbein J, et al. Quantitative Western ligand blotting reveals common patterns and differential features of IGFBP-fingerprints in domestic ruminant breeds and species. Growth Horm IGF Res. 2016;26:42–9. doi: 10.1016/j.ghir.2015.11.001 26597140

[23] Schregel J, Schulze Holthausen J, Sciascia M, Li Z, Görs S, Eggert A, et al. Effects of oral glutamine supplementation on jejunal morphology, development, and amino acid profiles in male low birth weight suckling piglets. PLoS One. 2022;17(4):e0267357. doi: 10.1371/journal.pone.0267357 35476806 PMC9045636

[24] Taylor S, Wakem M, Dijkman G, Alsarraj M, Nguyen M. A practical approach to RT-qPCR-Publishing data that conform to the MIQE guidelines. Methods. 2010;50(4):S1-5. doi: 10.1016/j.ymeth.2010.01.005 20215014

[25] Center for Drug E R ib, Center for Biologics E R ib. M10: bioanalytical method validation and study sample analysis: Guidance for industry. Silver Spring, MD: Center for Drug Evaluation and Research. 2022.

[26] Tian Q, Greig EE, Walker KA, Fishbein KW, Spencer RG, Resnick SM, et al. Plasma metabolomic markers underlying skeletal muscle mitochondrial function relationships with cognition and motor function. Age Ageing. 2024;53(4):afae079. doi: 10.1093/ageing/afae079 38615247 PMC11484644

[27] Rohart F, Gautier B, Singh A, Lê Cao K-A. mixOmics: An R package for ’omics feature selection and multiple data integration. PLoS Comput Biol. 2017;13(11):e1005752. doi: 10.1371/journal.pcbi.1005752 29099853 PMC5687754

[28] Pang Z, Lu Y, Zhou G, Hui F, Xu L, Viau C, et al. MetaboAnalyst 6.0: towards a unified platform for metabolomics data processing, analysis and interpretation. Nucleic Acids Res. 2024;52(W1):W398–406. doi: 10.1093/nar/gkae253 38587201 PMC11223798

[29] Worley B, Powers R. Multivariate analysis in metabolomics. Curr Metabolomics. 2013;1(1):92–107. doi: 10.2174/2213235X11301010092 26078916 PMC4465187

[30] Stone RT, Christenson RK. The relationship of fetal weight to serum albumin and alpha-fetoprotein in swine. J Anim Sci. 1982;55(4):818–25. doi: 10.2527/jas1982.554818x 6183246

[31] Ayala L, Sánchez CJ, Hernández F, Madrid J, López MJ, Martínez-Miró S. A comparison of haematological and biochemical profiles between intrauterine growth restriction and normal piglets at 72 hours postpartum. Animals (Basel). 2023;13(22):3540. doi: 10.3390/ani13223540 38003158 PMC10668781

[32] Vodolazska D, Feyera T, Lauridsen C. The impact of birth weight, birth order, birth asphyxia, and colostrum intake per se on growth and immunity of the suckling piglets. Sci Rep. 2023;13(1):8057. doi: 10.1038/s41598-023-35277-3 37198433 PMC10192303

[33] Szymeczko R, Kapelański W, Piotrowska A, Dybała J, Bogusławska-Tryk M, Burlikowska K, et al. Changes in the content of major proteins and selected hormones in the blood serum of piglets during the early postnatal period. Folia Biol (Krakow). 2009;57(1–2):97–103. doi: 10.3409/fb57_1-2.97-103 19459467

[34] Quesnel H, Resmond R, Merlot E, Père M-C, Gondret F, Louveau I. Physiological traits of newborn piglets associated with colostrum intake, neonatal survival and preweaning growth. Animal. 2023;17(6):100843. doi: 10.1016/j.animal.2023.100843 37263133

[35] Rajaram S, Baylink DJ, Mohan S. Insulin-like growth factor-binding proteins in serum and other biological fluids: regulation and functions. Endocr Rev. 1997;18(6):801–31. doi: 10.1210/edrv.18.6.0321 9408744

[36] Nissen PM, Nebel C, Oksbjerg N, Bertram HC. Metabolomics reveals relationship between plasma inositols and birth weight: Possible markers for fetal programming of type 2 diabetes. J Biomed Biotechnol. 2011;2011:378268. doi: 10.1155/2011/378268 20814537 PMC2931387

[37] López-Gambero AJ, Sanjuan C, Serrano-Castro PJ, Suárez J, Rodríguez de Fonseca F. The biomedical uses of inositols: A nutraceutical approach to metabolic dysfunction in aging and neurodegenerative diseases. Biomedicines. 2020;8(9):295. doi: 10.3390/biomedicines8090295 32825356 PMC7554709

[38] Metges CC, Görs S, Lang IS, Hammon HM, Brüssow K-P, Weitzel JM, et al. Low and high dietary protein: Carbohydrate ratios during pregnancy affect materno-fetal glucose metabolism in pigs. J Nutr. 2014;144(2):155–63. doi: 10.3945/jn.113.182691 24353346

[39] Uddin MK, Hasan S, Peltoniemi O, Oliviero C. The effect of piglet vitality, birth order, and blood lactate on the piglet growth performances and preweaning survival. Porcine Health Manag. 2022;8(1):52. doi: 10.1186/s40813-022-00299-2 36564789 PMC9789625

[40] Li W, Zhong X, Zhang L, Wang Y, Wang T. Heat shock protein 70 expression is increased in the liver of neonatal intrauterine growth retardation piglets. Asian-Australas J Anim Sci. 2012;25(8):1096–101. doi: 10.5713/ajas.2012.12058 25049668 PMC4092995

[41] Bhutia YD, Ganapathy V. Glutamine transporters in mammalian cells and their functions in physiology and cancer. Biochim Biophys Acta. 2016;1863(10):2531–9. doi: 10.1016/j.bbamcr.2015.12.017 26724577 PMC4919214

[42] Boldyrev AA, Aldini G, Derave W. Physiology and pathophysiology of carnosine. Physiol Rev. 2013;93(4):1803–45. doi: 10.1152/physrev.00039.2012 24137022

[43] Wu G, Knabe DA. Arginine synthesis in enterocytes of neonatal pigs. Am J Physiol. 1995;269(3 Pt 2):R621-9. doi: 10.1152/ajpregu.1995.269.3.R621 7573565

[44] Lu SC. Glutathione synthesis. Biochim Biophys Acta. 2013;1830(5):3143–53. doi: 10.1016/j.bbagen.2012.09.008 22995213 PMC3549305

[45] Tang X, Xiong K. Intrauterine growth retardation affects intestinal health of suckling piglets via altering intestinal antioxidant capacity, glucose uptake, tight junction, and immune responses. Oxid Med Cell Longev. 2022;2022:2644205. doi: 10.1155/2022/2644205 35345830 PMC8957421

[46] Chen J, Song Y, Chen D, Yu B, He J, Mao X. Low birth weight disturbs the intestinal redox status and mitochondrial morphology and functions in newborn piglets. Animals. 2021;11(9). doi: 10.3390/ani11092561 34573527 PMC8469446

[47] Bröer S. Amino acid transport across mammalian intestinal and renal epithelia. Physiol Rev. 2008;88(1):249–86. doi: 10.1152/physrev.00018.2006 18195088

[48] Jin S, Xu H, Yang C, O K. Regulation of oxidative stress in the intestine of piglets after enterotoxigenic Escherichia coli (ETEC) infection. Biochim Biophys Acta Mol Cell Res. 2024;1871(5):119711. doi: 10.1016/j.bbamcr.2024.119711 38574824

[49] Tang X, Xiong K, Fang R, Li M. Weaning stress and intestinal health of piglets: A review. Front Immunol. 2022;13:1042778. doi: 10.3389/fimmu.2022.1042778 36505434 PMC9730250

[50] Shcherbik N, Pestov DG. The impact of oxidative stress on ribosomes: from injury to regulation. Cells. 2019;8(11):1379. doi: 10.3390/cells8111379 31684095 PMC6912279

[51] Xiao YP, Wu TX, Sun JM, Yang L, Hong QH, Chen AG, et al. Response to dietary L-glutamine supplementation in weaned piglets: A serum metabolomic comparison and hepatic metabolic regulation analysis. J Anim Sci. 2012;90(12):4421–30. doi: 10.2527/jas.2012-5039 23255817

[52] Wu G, Knabe DA, Yan W, Flynn NE. Glutamine and glucose metabolism in enterocytes of the neonatal pig. Am J Physiol. 1995;268(2 Pt 2):R334–42. doi: 10.1152/ajpregu.1995.268.2.R334 7864226

[53] Xiao Y, Wu T, Hong Q, Sun J, Chen A, Yang C, et al. Response to weaning and dietary L-glutamine supplementation: metabolomic analysis in piglets by gas chromatography/mass spectrometry. J Zhejiang Univ Sci B. 2012;13(7):567–78. doi: 10.1631/jzus.B1100343 22761248 PMC3390714

[54] Liu Y. Fatty acids, inflammation and intestinal health in pigs. J Anim Sci Biotechnol. 2015;6(1):41. doi: 10.1186/s40104-015-0040-1 26361542 PMC4564983

[55] Fernández-Reina A, Urdiales JL, Sánchez-Jiménez F. What we know and what we need to know about aromatic and cationic biogenic amines in the gastrointestinal tract. Foods. 2018;7(9):145. doi: 10.3390/foods7090145 30181486 PMC6164962

