# Supplementary material for: Relationship between neonatal glutamine supplementation and amino acid profiles, protein synthesis in splanchnic tissues and the plasma metabolome in 5-day-old piglets with low birth weight
Source: PLoS One. 2026 Oct 12;21(10):e0359788. doi: 10.1371/journal.pone.0359788 (PMC13657303; doi:10.1371/journal.pone.0359788)
Supplement: Supplementary file 1 [file pone.0359788.s001.docx]

**S1 Fig. Diagnostic plot from DIABLO multiblock sparse partial least square discriminant analysis of duodenal (A) and liver (B) datasets from low (LBiW) and normal (NBiW) birthweight (BiW) piglets supplemented with glutamine or alanine from 24 hours after birth to 5 days of age, showing the correlation between components from each dataset ^1^.**

^1^ Samples are colored by experimental group (purple, LBiW-Ala; green, LBiW-Gln; black, NBiW-Ala; blue, NBiW-Gln) and 95% confidence ellipse plots are represented.

Abbreviations: AA, amino acids; FAA, free AA; PBAA, protein-bound AA.
