# Supplementary material for: Relationship between neonatal glutamine supplementation and amino acid profiles, protein synthesis in splanchnic tissues and the plasma metabolome in 5-day-old piglets with low birth weight
Source: PLoS One. 2026 Oct 12;21(10):e0359788. doi: 10.1371/journal.pone.0359788 (PMC13657303; doi:10.1371/journal.pone.0359788)
Supplement: Supplementary file 2 [file pone.0359788.s002.docx]

**S1 Table. Characteristics of experimental litters selected for the study**.

| **Litter characteristic^1^** | **Mean** | **SE** |
| --- | --- | --- |
| Farrowing duration (h:min) | 04:11 | 0:02 |
| Number of litters | 16 | |
| Number of piglets (total live + stillborn) | 256 | |
| Stillborn (total) | 8 | |
| No. of females (total) | 131 | |
| No. of males (total) | 117 | |
| Litter size at birth (average) | 16.00 | 0.32 |
| Live born females (per litter) | 8.19 | 0.63 |
| Live born males (per litter) | 7.31 | 0.66 |
| Bodyweight (kg) |  |  |
| Litter (average) | 19.44 | 0.58 |
| Piglet (average) | 1.24 | 0.02 |
| Female (average) | 1.22 | 0.02 |
| Males (average) | 1.26 | 0.02 |
| Intra-litter variability (kg)^2^ | 0.07 | 0.001 |
|  |  |  |

^1^Piglets born alive or dead (mummies excluded).

^2^Intra-litter variability of birth weight was calculated as square root of the pooled litter variance of male and female piglets within each litter.
