# Supplementary material for: Relationship between neonatal glutamine supplementation and amino acid profiles, protein synthesis in splanchnic tissues and the plasma metabolome in 5-day-old piglets with low birth weight
Source: PLoS One. 2026 Oct 12;21(10):e0359788. doi: 10.1371/journal.pone.0359788 (PMC13657303; doi:10.1371/journal.pone.0359788)
Supplement: Supplementary file 4 [file pone.0359788.s004.docx]

**S3 Table.** **Real time PCR primer sequences and product size for target mRNA in duodenal mucosa and liver tissue.**

| **mRNA^1^** | **Ref Seq No.** | **Primer sequence (5' - 3')** | **Length^2^** |
| --- | --- | --- | --- |
| **Amino acid transport** | | | |
| *SLC1A4*^a^ | XM_021087450.1 | **F:** AGCAGGACAGATTTTCACCATC | 149 |
|  |  | **R:** CCACAGCCAGGATCAAAGAG |  |
| *SLC38A3*^a^ | XM_003358448.4 | **F:** CTTCCTACAGAAAAGCCCCAG | 92 |
|  |  | **R:** CGTTGCTAAGGTTGAACACTG |  |
| *SLC38A4*^a^ | XM_021092580.1 | **F:** AGAGCATCAGTGGAGAAAGTG | 104 |
|  |  | **R:** TTGACTTTCAGCATCTTCGTTTG |  |
| *SLC6A19*^b^ | XM_003359855.4 | **F:** CCGTTGATAAGCGTCAGGAT | 155 |
|  |  | **R:** CACAACAACTGCGAGAAGGA |  |
| *SLC7A8*^a^ | XM_021099239.1 | **F:** AATCTTCGTCTCACCCAAAGG | 110 |
|  |  | **R:** CATAACACAGGGCTCCTACAG |  |
| **Glutamine metabolism** | | | |
| *AST1*^a^ | NM_213927.1 | **F:** CTTGCCCTTGGAGATGACAG | 120 |
|  |  | **R:** TCCATTGTACCATCGTGCTAAG |  |
| *AST2*^a^ | NM_213928.1 | **F:** TGGGTGAGAACAATGAGGTG | 137 |
|  |  | **R:** TTTCGGCAGGAAGACATCTC |  |
| *GLUD1*^a^ | NM_001244501.2 | **F:** TCCTGGGAGGTTATCGAGG | 101 |
|  |  | **R:** ACTTCATCTACGCTCACATCAG |  |
| *GLS*^a^ | XM_021076054.1 | **F:** CAGTTTGATGCATTCATGTGG | 78 |
|  |  | **R:** CCAGATTTTGCAGGAAGACC |  |
| *NAGS*^a^ | NM_001097520.1 | **F:** ACTCTGTCTACGTCTCTGAGG | 93 |
|  |  | **R:** CCACAAACTTGTCTAGATACGGG |  |
| **β-oxidation** | | | |
| *ACAA2*^c^ | NM_001167638.1 | **F:** GGCACTGAAGAAAGCAGGAC | 238 |
|  |  | **R:** GGCTGATCCCACAGCATATT |  |
| *ACACA*^c^ | NM_001114269.1 | **F:** GGTTATGTGAAGGATGTGGATG | 75 |
|  |  | **R:** TGAGGCCTTGATCATTACTGG |  |
| *ACADL*^c^ | NM_213897.1 | **F:** CATGAAACGAAACGTCTGGA | 159 |
|  |  | **R:** AACTCGGGCATCCACATAAG |  |
| *CPT1A*^c^ | NM_001129805.1 | **F:** AAGGTGCTGCTCTCCTACCA | 204 |
|  |  | **R:** CATCAGAGGCTTCACGGATT |  |
| *HADH*^c^ | NM_214331.1 | **F:** GAGGAAAGCCTTCGGAAAGT | 175 |
|  |  | **R:** CGCTCTTCACCTTCAGGTTC |  |
| **Lipid synthesis** | | | |
| *DGAT1*^c^ | NM_214051.1 | **F:** GGAGATGCTGTTCCTCATCC | 87 |
|  |  | **R:** TTGAAGGGCTTCATGGAGTT |  |
| *DGAT2*^c^ | NM_001160080.1 | **F:** CCAGCAGGTGATCTTTGAGG | 76 |
|  |  | **R:** GGCGAAGCCAATGTACTTCT |  |
| *FABP3*^c^ | NM_001099931.1 | **F:** AAGCACCTTCAAGAGCACAGA | 92 |
|  |  | **R:** TCACAATGGACTTGACCTTCC |  |
| *SCD*^c^ | NM_213781.1 | **F:** CTGGCTTATGACCGGAAGAA | 96 |
|  |  | **R:** ACCCCAAACTCAGCCACTC |  |
| **Transcription factors** | | | |
| *PGC-1*^c^ | NM_213963.1 | **F:** CCGAGAATTCATGGAGCAAT | 162 |
|  |  | **R:** TGGAGGTGCACTTGTCTCTG |  |
| *PPARA*^c^ | NM_001044526.1 | **F:** TGCCAGTATTGTCGTTTCCA | 219 |
|  |  | **R:** GGCCTTGACCTTGTTCATGT |  |
| **Glutathione synthesis** | | | |
| *GCLC*^d^ | XM_021098555.1 | **F:** TGATCCCCATCCTGAACTCT | 194 |
|  |  | **R:** CGGATGGTTTGCGATAAACT |  |
| *CGL*^e^ | NM_001044585.2 | **F:** CTTCATAGTAGGCTCCGTTT | 142 |
|  |  | **R:** ACTGAGCAACTGCCATTCCA |  |
| *GGT*^d^ | XM_021072100.1 | **F:** CAAAGGGTACAACTTCTCC | 338 |
|  |  | **R:** AGCCAAAGTAGAGGTTGAT |  |
| *GPX1*^a^ | NM_214201.1 | **F:** GGGACTACACCCAGATGAATG | 92 |
|  |  | **R:** CTGATGTCCAAACTGGTTGC |  |
| *GPX2*^a^ | NM_001115136.1 | **F:** CCTGGTGGTTCTTGGCTTC | 74 |
|  |  | **R:** TGTTCAGGATCTCCTCATTCTG |  |
| *GPX4*^a^ | NM_214407.1 | **F:** ACGAATTCTCAGCCAAGGAC | 96 |
|  |  | **R:** CTCATTGAGAGGCCACATTG |  |
| *GSR*^a^ | XM_003483635.4 | **F:** ATGTCGGATGTGTACCCAAA | 82 |
|  |  | **R:** CTCTCACAACTCTGAAAGCCA |  |
| *GSS*^d^ | NM_001244625.1 | **F:** GTGCTCAAGCCCCAGAGA | 100 |
|  |  | **R:** ATGAGGCTCTCTCCTCACTGTC |  |
| *GSTA1*^a^ | NM_213850.2 | **F:** CCGTTATCTTCCTGCATTTG | 82 |
|  |  | **R:** GTCAGCCCTGCTCAGCTT |  |
| *OPLAH*^d^ | XM_013988002 | **F:** CAACCCGAGTCAGGGTAGC | 111 |
|  |  | **R:** AGGCAGTAGATGAGGGCAGA |  |
| **Tight junction proteins** | | | |
| *CLDN4*^a^ | NM_001161637.1 | **F:** CAACTGCGTGGATGATGAGA | 140 |
|  |  | **R:** CCAGGGGATTGTAGAAGTCG |  |
| *TJP1*^a^ | XM_021098827.1 | **F:** GACCAACGTAGCTCTGGCAT | 192 |
|  |  | **R:** TGGACATAGCCTCATTCGCA |  |
| *TJP2*^a^ | XM_005660143.3 | **F:** TAAAGATGGCAACCTGCACGA | 155 |
|  |  | **R:** TGATGAGCGTCTGCTTGCTG |  |
| **Miscellaneous** | | | |
| *CASP3*^e^ | NM_214131.1 | **F:** GAACTCTAACTGGCAAACCCA | 105 |
|  |  | **R:** ACGCCATGTCATCTTCAGTC |  |
| *SDHA*^f^ | XM_021076931.1 | **F:** GCGGACAGAGCCTCAAGTT | 155 |
|  |  | **R:** CAGGAGATCCAAGGCAAAAT |  |
| *SOD1*^d^ | GU944822.1 | **F:** GAGACCTGGGCAATGTGACT | 139 |
|  |  | **R:** CTGCCCAAGTCATCTGGTTT |  |

^1^ Abbreviations: **ACAA2,** Acetyl-CoA acyltransferase 2; **ACACA**, Acetyl-CoA carboxylase Alpha; **ACADL**, Acyl-CoA dehydrogenase. long chain; **AST1/2**, Aspartate-amino-Transferase 1/2; **CASP3**, Caspase-3; **CLDN4**, Claudin 4; **CPT1A**, Carnitine palmitoyltransferase 1A; **DGAT1/2**, Diacylglycerol acyltransferase 1/2; **FABP3**, Fatty acid-binding protein 3; **GCLC**, Glutamate-cysteine ligase catalytic subunit; **CGL**, Cystathionine Gamma-Lyase; **GGT**, Gamma-Glutamyltransferase 1; **GLS**, Glutaminase; **GLUD1**, Glutamate dehydrogenase; **GPX1/2/4**, Glutathione Peroxidase 1/2/4; **GSR**, Glutathione Reductase, Mitochondrial; **GSS**, Glutathione Synthetase; **GSTA1**, Glutathione S-Transferase Alpha 1; **HADH**, Hydroxyacyl-CoA dehydrogenase; **NAGS**, N-Acetylglutamate Synthase; **OPLAH**, 5-Oxoprolinase; **PGC-1**, Peroxisome Proliferator-Activated Receptor Gamma Coactivator 1-Alpha; **PPARA**, Peroxisome proliferator-activated receptor alpha; **SCD**, Stearoyl-CoA desaturase; **SDHA**, Succinate dehydrogenase complex, subunit A; **SLC**, Solute Linked Carrier 1A4; 38A3; 38A4; 6A19; 7A8; **TJP1/2**, Tight Junction Protein 1/2; **SOD1**, Superoxide Dismutase 1.

^a^ Primer sourced from [5].

^b^ Primer sourced from [6].

^c^ Primer sourced from [2].

^d^ Primer sourced from [7].

^e^ Designed for this study.

^f^ Primer sourced from [3].

# Literature Cited

1.	Nygard AB, Jorgensen CB, Cirera S, Fredholm M. Selection of reference genes for gene expression studies in pig tissues using SYBR green qPCR. BMC Mol Biol. 2007;8:67. Epub 20070815. doi: 10.1186/1471-2199-8-67. PubMed PMID: 17697375; PubMed Central PMCID: PMCPMC2000887.

2.	Sciascia M, Das G, Maak S, Kalbe C, Metzler-Zebeli BU, Metges CC. Transcript profile of skeletal muscle lipid metabolism genes affected by diet in a piglet model of low birth weight. PLoS One. 2019;14(10):e0224484. Epub 20191029. doi: 10.1371/journal.pone.0224484. PubMed PMID: 31661531; PubMed Central PMCID: PMCPMC6818798.

3.	Feng X, Xiong Y, Qian H, Lei M, Xu D, Ren Z. Selection of reference genes for gene expression studies in porcine skeletal muscle using SYBR green qPCR. J Biotechnol. 2010;150(3):288-93. Epub 20100929. doi: 10.1016/j.jbiotec.2010.09.949. PubMed PMID: 20887758.

4.	Zhang J, Tang Z, Wang N, Long L, Li K. Evaluating a set of reference genes for expression normalization in multiple tissues and skeletal muscle at different development stages in pigs using quantitative real-time polymerase chain reaction. DNA Cell Biol. 2012;31(1):106-13. Epub 20110601. doi: 10.1089/dna.2011.1249. PubMed PMID: 21631284.

5.	Schregel J, Schulze Holthausen J, Sciascia M, Gors S, Li Z, Tuchscherer A, et al. Acute and persistent effects of oral glutamine supplementation on growth, cellular proliferation, and tight junction protein transcript abundance in jejunal tissue of low and normal birthweight pre-weaning piglets. PLoS One. 2024;19(1):e0296427. Epub 20240102. doi: 10.1371/journal.pone.0296427. PubMed PMID: 38165864; PubMed Central PMCID: PMCPMC10760696.

6.	Yang C, Yang X, Lackeyram D, Rideout TC, Wang Z, Stoll B, et al. Expression of apical Na(+)-L-glutamine co-transport activity, B(0)-system neutral amino acid co-transporter (B(0)AT1) and angiotensin-converting enzyme 2 along the jejunal crypt-villus axis in young pigs fed a liquid formula. Amino Acids. 2016;48(6):1491-508. Epub 20160316. doi: 10.1007/s00726-016-2210-7. PubMed PMID: 26984322.

7.	Rasch I, Gors S, Tuchscherer A, Viergutz T, Metges CC, Kuhla B. Substitution of Dietary Sulfur Amino Acids by dl-2-Hydroxy-4-Methylthiobutyric Acid Reduces Fractional Glutathione Synthesis in Weaned Piglets. J Nutr. 2020;150(4):722-9. doi: 10.1093/jn/nxz272. PubMed PMID: 31773161; PubMed Central PMCID: PMCPMC7138682.
