# Supplementary material for: Relationship between neonatal glutamine supplementation and amino acid profiles, protein synthesis in splanchnic tissues and the plasma metabolome in 5-day-old piglets with low birth weight
Source: PLoS One. 2026 Oct 12;21(10):e0359788. doi: 10.1371/journal.pone.0359788 (PMC13657303; doi:10.1371/journal.pone.0359788)
Supplement: Supplementary file 5 [file pone.0359788.s005.docx]

**S4 Table.** **List of variables included in the DIABLO analysis.**

| **Blocks and Variables** | **Abbreviation** |
| --- | --- |
| **Liver and Duodenum Block 1:** Free amino acids**,** *n* = 20 |  |
| **Liver and Duodenum Block 3:** Protein-bound amino acids**,** *n* = 20 |  |
| Alanine | Ala |
| Arginine | Arg |
| Asparagine | Asn |
| Aspartate | Asp |
| Cysteine | Cys |
| Glutamine | Gln |
| Glutamate | Glu |
| Glycine | Gly |
| Histidine | His |
| Isoleucine | Ile |
| Leucine | Leu |
| Lysine | Lys |
| Methionine | Met |
| Phenylalanine | Phe |
| Proline | Pro |
| Serine | Ser |
| Threonine | Thr |
| Tryptophan | Trp |
| Tyrosine | Tyr |
| Valine | Val |
| **Liver Block 2:** AA-metabolites, *n* = 12 | |
| 1-Methylhistidine | 1-MH |
| 3-Methylhistidine | 3-MH |
| Alpha-aminoadipic acid | Aad |
| Alpha-aminobutyric acid | Abu |
| α-Aminoisobutyric acid | Aib |
| Beta-alanine | bAla |
| Carnosine | Car |
| Citrulline | Cit |
| Gamma-aminobutyric acid | GABA |
| Hydroxyproline | HyP |
| Ornithine | Orn |
| Taurine | Tau |
| **Liver Block 4:** mRNA, *n* = 29 | |
| Acetyl-CoA Acyltransferase 2 | ACAA2 |
| Acetyl-CoA Carboxylase Alpha | ACACA |
| Acyl-CoA Dehydrogenase Long Chain | ACADL |
| Aspartate Transaminase 1 | AST1 |
| Aspartate Transaminase 2 | AST2 |
| Caspase-3 | CASP3 |
| Cystathionine Gamma-Lyase | CGL |
| Carnitine Palmitoyltransferase 1A | CPT1A |
| Diacylglycerol O-Acyltransferase 1 | DGAT1 |
| Diacylglycerol O-Acyltransferase 2 | DGAT2 |
| Fatty Acid Binding Protein 3 | FABP3 |
| Glutaminase | GLS |
| Glutamate Dehydrogenase 1 | GLUD1 |
| Glutathione Peroxidase 1 | GPX1 |
| Glutathione-Disulfide Reductase | GSR |
| Glutathione S-Transferase Alpha 1 | GSTA1 |
| Hydroxyacyl-CoA Dehydrogenase | HADH |
| Hypoxanthine Phosphoribosyltransferase 1 | HPRT |
| Proliferating Cell Nuclear Antigen | PCNA |
| Peroxisome Proliferator-Activated Receptor 1 | PPARA1 |
| Peroxisome Proliferative Activated Receptor, Gamma, Coactivator 1 | PPARCG1 |
| Ribosomal Protein S18 | RPS18 |
| Stearoyl-CoA Desaturase | SCD |
| Succinate Dehydrogenase Complex Flavoprotein Subunit A | SDHA |
| Solute Carrier Family 1 Member 4 | SLC1A4 |
| Solute Carrier Family 38 Member 3 | SLC38A3 |
| Solute Carrier Family 38 Member 4 | SLC38A4 |
| Superoxide Dismutase 1 | SOD1 |
| DNA Topoisomerase II Beta | TOP2B |
| **Duodenum Block 2:** Amino-metabolites, *n* = 10 | |
| Alpha-aminoadipic acid | Aad |
| Alpha-aminobutyric acid | Abu |
| α-Aminoisobutyric acid | Aib |
| Beta-alanine | bAla |
| Carnosine | Car |
| Citrulline | Cit |
| Gamma-aminobutyric acid | GABA |
| Hydroxyproline | HyP |
| Ornithine | Orn |
| Taurine | Tau |
| **Duodenum Block 4:** mRNA**,** *n* = 26 | |
| Beta-Actin | ACTB |
| Aspartate Transaminase 1 | AST1 |
| Aspartate Transaminase 2 | AST2 |
| Caspase-3 | CASP3 |
| Cystathionine Gamma-Lyase | CGL |
| Claudin 4 | CLDN4 |
| Glutamate-cysteine ligase catalytic subunit | GCLC |
| Gamma-Glutamyltransferase 1 | GGT |
| Glutaminase | GLS |
| Glutamate Dehydrogenase 1 | GLUD1 |
| Glutathione Peroxidase 1 | GPX1 |
| Glutathione Peroxidase 2 | GPX2 |
| Glutathione Peroxidase 4 | GPX4 |
| Glutathione-Disulfide Reductase | GSRa1 |
| Glutathione Synthetase | GSS |
| Glutathione S-Transferase Alpha 1 | GSTA1 |
| N-Acetylglutamate Synthase | NAGS |
| 5-Oxoprolinase, ATP-Hydrolysing | OPLAH |
| Proliferating Cell Nuclear Antigen | PCNA |
| Succinate Dehydrogenase Complex Flavoprotein Subunit A | SDHA |
| Solute Carrier Family 6 Member 19 | SLC6A19 |
| Solute Carrier Family 7 Member 8 | SLC7A8 |
| Superoxide Dismutase 1 | SOD1 |
| DNA Topoisomerase II Beta | TOP2B |
| Tight Junction Protein 1 | TJP1 |
| Tight Junction Protein 2 | TJP2 |
