# Supplementary material for: Relationship between neonatal glutamine supplementation and amino acid profiles, protein synthesis in splanchnic tissues and the plasma metabolome in 5-day-old piglets with low birth weight
Source: PLoS One. 2026 Oct 12;21(10):e0359788. doi: 10.1371/journal.pone.0359788 (PMC13657303; doi:10.1371/journal.pone.0359788)
Supplement: Supplementary file 6 [file pone.0359788.s006.docx]

**S5 Table.** **Bodyweight gain, and concentrations of amino acid and clinical-chemistry parameters in plasma of low (LBiW) and normal (NBiW) birthweight piglets at birth (0 h).**

| **Item^1,4^** | **LBiW** | **NBiW** | ***P-*values** |
| --- | --- | --- | --- |
| Bodyweight |  |  |  |
| Bodyweight gain, %^2,3^ | 0.64 ± 1.10 | 1.55 ± 1.02 | 0.368 |
| Amino acids, µmol/L |  |  |  |
| **Indispensable AA** | 1955.51 ± 218.95 | 2176.40 ± 229.88 | 0.365 |
| Arginine | 169.32 ± 23.91 | 179.36 ± 25.33 | 0.747 |
| Histidine | 136.46 ± 13.94 | 144.44 ± 14.44 | 0.510 |
| Isoleucine | 91.08 ± 13.17 | 111.51 ± 13.86 | 0.185 |
| Leucine | 254.14 ± 36.94 | 304.49 ± 38.57 | 0.185 |
| Lysine | 477.14 ± 45.80 | 513.78 ± 48.49 | 0.532 |
| Methionine | 68.24 ± 11.18 | 63.86 ± 12.44 | 0.760 |
| Phenylalanine | 124.18 ± 13.51 | 159.01 ± 13.93 | 0.077 |
| Threonine | 204.80 ± 23.57 | 228.13 ± 24.89 | 0.423 |
| Tryptophan | 30.70 ± 2.34 | 35.10 ± 2.41 | 0.180 |
| Valine | 387.76 ± 38.45 | 415.08 ± 40.98 | 0.507 |
| **Dispensable AA** | 5653.01 ± 405.90 | 5867.91 ± 427.39 | 0.648 |
| Alanine | 630.50 ± 42.54 | 720.14 ± 43.47 | 0.083 |
| Asparagine | 131.94 ± 13.11 | 144.19 ± 13.84 | 0.443 |
| Aspartate | 87.57 ± 9.73 | 85.57 ± 10.33 | 0.881 |
| Glutamate | 1024.18 ± 84.55 | 976.70 ± 90.01 | 0.705 |
| Glutamine | 803.76 ± 39.79 | 781.20 ± 42.35 | 0.701 |
| Glycine | 1097.34 ± 75.01 | 1169.29 ± 76.76 | 0.430 |
| Proline | 1078.37 ± 111.04 | 1159.81 ± 128.07 | 0.592 |
| Serine | 348.48 ± 28.88 | 400.22 ± 29.61 | 0.165 |
| Tyrosine | 235.97 ± 31.68 | 260.11 ± 33.27 | 0.495 |
| Total AA | 8531.36 ± 660.62 | 9018.25 ± 694.64 | 0.517 |
| 1-MH | 14.28 ± 0.91 | 14.68 ± 0.94 | 0.767 |
| 2-MH | 13.94 ± 0.77 | 14.14 ± 0.82 | 0.861 |
| Aad | 46.49 ± 5.10 | 53.85 ± 5.45 | 0.337 |
| Aib | 2.12 ± 0.10 | 2.17 ± 0.10 | 0.629 |
| Anserine | 1.46 ± 0.08 | 1.57 ± 0.08 | 0.109 |
| Beta-alanine | 15.95 ± 1.32 | 16.32 ± 1.41 | 0.833 |
| Carnosine | 21.82 ± 1.46 | 20.71 ± 1.54 | 0.529 |
| Citrulline | 160.90 ± 12.87 | 143.81 ± 14.27 | 0.299 |
| GABA | 2.40 ± 0.12 | 2.28 ± 0.14 | 0.531 |
| HyP | 123.00 ± 6.63 | 122.34 ± 6.8 | 0.939 |
| Ornithine | 86.37 ± 9.05 | 98.17 ± 9.63 | 0.366 |
| Taurine | 133.05 ± 17.33 | 172.53 ± 17.71 | 0.063 |
| **Clinical parameters** |  |  |  |
| AST (U/L) | 89.25 ± 7.60 | 83.56 ± 8.57 | 0.527 |
| AST to ALT ratio | 2.90 ± 0.21 | 3.47 ± 0.22 | 0.057 |
| Cholesterol (mmol/L) | 1.51 ± 0.09 | 1.56 ± 0.09 | 0.556 |
| Fructose (mmol/L) | 2.02 ± 0.11 | 2.11 ± 0.11 | 0.398 |
| NEFA (µmol/L) | 175.17 ± 20.80 | 200.67 ± 20.80 | 0.081 |
| Triglycerides (mmol/L) | 0.45 ± 0.05 | 0.48 ± 0.05 | 0.296 |
| Urea (µmol/L) | 4.20 ± 0.25 | 4.29 ± 0.25 | 0.495 |
| IGFBP-2 | 2999.54 ± 184.78 | 2910.06 ± 181.53 | 0.585 |
| IGFBP-5 | 404.91 ± 29.80 | 375.32 ± 29.37 | 0.383 |

^1^ Values are LSM ± SEM; *n* = 48, *n* = 24 / subgroup.

^2^ *n* = 41, *n* = 21 LBiW, *n* = 20 NBiW.

^4^ Bodyweight gain, % = ((Bodyweight at 4h post birth, kg - Birthweight, kg) / Birthweight, kg)) × 100

^3^ Abbreviations: 1-MH, 1-methylhistidine; 2-MH, 2-methylhistidine; AA, amino acid; Aad, alpha-aminoadipic acid; Aib, α-aminoisobutyric acid; AST, aspartate transaminase; ALT, alanine transaminase; GABA, gamma-aminobutyric acid; HyP, hydroxyproline; IGFBP-2/IGFBP-5, insulin-like growth factor binding protein 2 and 5; NEFA, non-esterified fatty acids.
