# Supplementary material for: Relationship between neonatal glutamine supplementation and amino acid profiles, protein synthesis in splanchnic tissues and the plasma metabolome in 5-day-old piglets with low birth weight
Source: PLoS One. 2026 Oct 12;21(10):e0359788. doi: 10.1371/journal.pone.0359788 (PMC13657303; doi:10.1371/journal.pone.0359788)
Supplement: Supplementary file 7 [file pone.0359788.s007.docx]

**S6 Table.** **Stomach content of free amino acid and amino acid (AA) metabolite profiles in low (LBiW) and normal (NBiW) birthweight (BiW) piglets supplemented with glutamine or alanine from 24 h after birth to 5 d of age.**

| **Item^1,2^** | **LBiW** | | **NBiW** | | ***P*-values^3^** | | |
| --- | --- | --- | --- | --- | --- | --- | --- |
|  | **Alanine** | **Glutamine** | **Alanine** | **Glutamine** | **BiW** | **Suppl** | **Interaction** |
| **AA** |  |  |  |  |  |  |  |
| Arginine | 0.15 ± 0.07 | 0.08 ± 0.01 | 0.13 ± 0.04 | 0.08 ± 0.01 | 0.974 | 0.159 | 0.610 |
| Histidine | 0.47 ± 0.13 | 0.39 ± 0.03 | 0.36 ± 0.08 | 0.35 ± 0.06 | 0.302 | 0.812 | 0.591 |
| Isoleucine | 0.23 ± 0.06 | 0.18 ± 0.04 | 0.28 ± 0.06 | 0.18 ± 0.04 | 0.568 | 0.308 | 0.848 |
| Leucine | 0.59 ± 0.14 | 0.56 ± 0.11 | 0.78 ± 0.14 | 0.62 ± 0.19 | 0.666 | 0.380 | 0.510 |
| Lysine | 0.50 ± 0.11 | 0.50 ± 0.07 | 0.60 ± 0.13 | 0.42 ± 0.10 | 0.646 | 0.241 | 0.464 |
| Phenylalanine | 0.47 ± 0.08 | 0.50 ± 0.10 | 0.57 ± 0.07 | 0.52 ± 0.14 | 0.708 | 0.548 | 0.572 |
| Threonine | 0.14 ± 0.06 | 0.09 ± 0.02 | 0.13 ± 0.05 | 0.11 ± 0.05 | 0.994 | 0.568 | 0.629 |
| Tryptophan | 0.12 ± 0.04 | 0.13 ± 0.03 | 0.13 ± 0.02 | 0.17 ± 0.04 | 0.166 | 0.399 | 0.595 |
| Valine | 0.15 ± 0.04 | 0.24 ± 0.04 | 0.20 ± 0.05 | 0.24 ± 0.05 | 0.412 | 0.199 | 0.864 |
| Alanine | 160.93 ± 74.98^a^ | 1.71 ± 0.37^b^ | 113.52 ± 16.57^a^ | 1.21 ± 0.23^b^ | 0.531 | 0.000 | 0.588 |
| Asparagine | 0.06 ± 0.01 | 0.06 ± 0.01 | 0.07 ± 0.01 | 0.08 ± 0.01 | 0.142 | 0.643 | 0.962 |
| Aspartate | 0.46 ± 0.04 | 0.68 ± 0.06 | 0.63 ± 0.12 | 0.66 ± 0.10 | 0.486 | 0.129 | 0.345 |
| Glutamate | 1.21 ± 0.34^b^ | 3.78 ± 0.68^a^ | 1.55 ± 0.44^b^ | 3.50 ± 0.71^a^ | 0.793 | 0.005 | 0.908 |
| Glutamine | 0.31 ± 0.05^b^ | 64.75 ± 20.83^a^ | 0.27 ± 0.03^b^ | 70.47 ± 31.18^a^ | 0.965 | 0.000 | 0.613 |
| Glycine | 0.65 ± 0.21 | 0.73 ± 0.13 | 1.00 ± 0.15 | 0.87 ± 0.13 | 0.065 | 0.921 | 0.490 |
| Serine | 0.43 ± 0.06 | 0.50 ± 0.07 | 0.55 ± 0.06 | 0.51 ± 0.06 | 0.328 | 0.417 | 0.399 |
| Tyrosine | 0.69 ± 0.14 | 0.62 ± 0.10 | 0.72 ± 0.10 | 0.49 ± 0.07 | 0.845 | 0.702 | 0.553 |
| Citrulline | 0.05 ± 0.02 | 0.07 ± 0.01 | 0.06 ± 0.01 | 0.08 ± 0.03 | 0.829 | 0.758 | 0.672 |
| GABA | 0.40 ± 0.19 | 0.38 ± 0.14 | 0.58 ± 0.24 | 0.71 ± 0.45 | 0.639 | 0.359 | 0.936 |
| Ornithine | 0.37 ± 0.05 | 0.58 ± 0.08 | 0.54 ± 0.09 | 0.60 ± 0.02 | 0.311 | 0.245 | 0.534 |
| Taurine | 0.71 ± 0.13 | 0.79 ± 0.03 | 0.89 ± 0.09 | 1.22 ± 0.33 | 0.050 | 0.113 | 0.877 |

^1^ Units: µmol/g stomach content dry matter. Values are raw means ± SE; *n* = 5 - 8 per BiW x Supplementation (Suppl) group. Interaction is the BiW x Suppl interaction.

^2^ Abbreviations: GABA, gamma-aminobutyric acid.

^3^ ANOVA *F* Test.

^a,b^ Different from Alanine-supplemented piglets within BiW group (*P* < 0.05).
