# Supplementary material for: Relationship between neonatal glutamine supplementation and amino acid profiles, protein synthesis in splanchnic tissues and the plasma metabolome in 5-day-old piglets with low birth weight
Source: PLoS One. 2026 Oct 12;21(10):e0359788. doi: 10.1371/journal.pone.0359788 (PMC13657303; doi:10.1371/journal.pone.0359788)
Supplement: Supplementary file 8 [file pone.0359788.s008.docx]

**S7 Table.** **Duodenal tissue free amino acid and amino acid (AA) metabolite profiles in low (LBiW) and normal (NBiW) birthweight (BiW) piglets supplemented with glutamine or alanine from 24 h after birth to 5 d of age.**

| **Item^1,2^** | **LBiW** | | **NBiW** | | | ***P*-values^3^** | | |
| --- | --- | --- | --- | --- | --- | --- | --- | --- |
|  | **Alanine** | **Glutamine** | | **Alanine** | **Glutamine** | **BiW** | **Suppl** | **Interaction** |
| **AA** |  |  | |  |  |  |  |  |
| **Indispensable AA** | 9.80 ± 4.02 | 5.12 ± 0.33 | | 6.48 ± 1.19 | 4.97 ± 0.38 | 0.200 | 0.627 | 0.741 |
| Arginine | 1.71 ± 1.08 | 0.57 ± 0.04 | | 0.90 ± 0.33 | 0.62 ± 0.04 | 0.608 | 0.164 | 0.313 |
| Histidine | 0.39 ± 0.10 | 0.30 ± 0.03 | | 0.32 ± 0.04 | 0.25 ± 0.03 | 0.439 | 0.333 | 0.554 |
| Isoleucine | 0.57 ± 0.17 | 0.35 ± 0.03 | | 0.41 ± 0.07 | 0.31 ± 0.04 | 0.106 | 0.152 | 0.843 |
| Leucine | 1.40 ± 0.58 | 0.68 ± 0.07 | | 0.84 ± 0.17 | 0.60 ± 0.06 | 0.081 | 0.175 | 0.685 |
| Lysine | 2.26 ± 1.21 | 1.00 ± 0.06 | | 1.44 ± 0.43 | 1.00 ± 0.08 | 0.467 | 0.354 | 0.694 |
| Methionine | 0.41 ± 0.16 | 0.22 ± 0.02 | | 0.27 ± 0.05 | 0.21 ± 0.02 | 0.260 | 0.249 | 0.601 |
| Phenylalanine | 0.89 ± 0.46 | 0.46 ± 0.04 | | 0.56 ± 0.08 | 0.44 ± 0.03 | 0.220 | 0.116 | 0.405 |
| Threonine | 1.22 ± 0.16^a^ | 0.95 ± 0.03^b^ | | 1.09 ± 0.07 | 1.03 ± 0.05 | 0.949 | 0.060 | 0.196 |
| Tryptophan | 0.14 ± 0.04 | 0.10 ± 0.01 | | 0.10 ± 0.02 | 0.09 ± 0.01 | 0.092 | 0.534 | 0.660 |
| Valine | 0.80 ± 0.21 | 0.50 ± 0.04 | | 0.57 ± 0.08 | 0.47 ± 0.05 | 0.120 | 0.150 | 0.576 |
| **Dispensable AA** | 34.40 ± 2.13^a^ | 27.73 ± 1.36^b^ | | 30.84 ± 1.43 | 27.21 ± 1.37 | 0.247 | 0.004 | 0.444 |
| Alanine | 10.46 ± 1.50^a^ | 3.23 ± 0.19^b^ | | 8.69 ± 1.21^a^ | 3.29 ± 0.16^b^ | 0.422 | 0.000 | 0.289 |
| Asparagine | 0.49 ± 0.08 | 0.39 ± 0.03 | | 0.38 ± 0.05 | 0.35 ± 0.03 | 0.112 | 0.534 | 0.677 |
| Aspartate | 1.85 ± 0.11 | 1.73 ± 0.08 | | 1.74 ± 0.08 | 1.76 ± 0.09 | 0.683 | 0.552 | 0.494 |
| Cysteine | 0.83 ± 0.07 | 0.68 ± 0.04 | | 0.73 ± 0.03 | 0.67 ± 0.04 | 0.341 | 0.039 | 0.562 |
| Glutamate | 6.58 ± 0.26 | 6.58 ± 0.30 | | 6.54 ± 0.27 | 6.56 ± 0.22 | 0.954 | 0.959 | 0.907 |
| Glutamine | 1.36 ± 0.21^a^ | 4.07 ± 0.45^b^ | | 1.13 ± 0.11^a^ | 3.95 ± 0.68^b^ | 0.307 | 0.000 | 0.908 |
| Glycine | 6.93 ± 0.26 | 6.57 ± 0.17 | | 6.95 ± 0.19 | 6.48 ± 0.16 | 0.979 | 0.060 | 0.703 |
| Proline | 3.48 ± 0.44 | 2.88 ± 0.24 | | 2.88 ± 0.31 | 2.33 ± 0.26 | 0.029 | 0.230 | 0.628 |
| Serine | 1.47 ± 0.22 | 1.08 ± 0.07 | | 1.21 ± 0.11 | 1.08 ± 0.09 | 0.326 | 0.150 | 0.428 |
| Tyrosine | 0.96 ± 0.37 | 0.52 ± 0.04 | | 0.59 ± 0.09 | 0.46 ± 0.04 | 0.081 | 0.236 | 0.656 |
| **Total AA** | 47.84 ± 5.46^a^ | 36.64 ± 1.79^b^ | | 41.07 ± 2.07 | 36.34 ± 1.71 | 0.284 | 0.028 | 0.389 |
| **AA-metabolites** |  |  | |  |  |  |  |  |
| Aad | 0.05 ± 0.004 | 0.06 ± 0.003 | | 0.05 ± 0.002 | 0.06 ± 0.002 | 0.917 | 0.075 | 0.553 |
| Abu | 0.04 ± 0.004 | 0.03 ± 0.004 | | 0.04 ± 0.002 | 0.03 ± 0.003 | 0.620 | 0.028 | 0.970 |
| Aib | 0.01 ± 0.001 | 0.01 ± 0.001 | | 0.01 ± 0.001 | 0.01 ± 0.001 | 0.266 | 0.667 | 0.434 |
| Beta-Alanine | 0.09 ± 0.01 | 0.08 ± 0.002 | | 0.08 ± 0.004 | 0.09 ± 0.01 | 0.935 | 0.817 | 0.033 |
| Carnosine | 0.04 ± 0.003^a^ | 0.05 ± 0.003^b^ | | 0.04 ± 0.003^a^ | 0.06 ± 0.004^b^ | 0.185 | 0.000 | 0.522 |
| Citrulline | 0.34 ± 0.04^a^ | 0.50 ± 0.05^b^ | | 0.35 ± 0.05^a^ | 0.47 ± 0.02^b^ | 0.955 | 0.001 | 0.849 |
| GABA | 0.14 ± 0.02 | 0.20 ± 0.04 | | 0.12 ± 0.02 | 0.14 ± 0.02 | 0.206 | 0.087 | 0.852 |
| HyP | 0.30 ± 0.02^d^ | 0.28 ± 0.01 | | 0.36 ± 0.02^c^ | 0.31 ± 0.01 | 0.017 | 0.103 | 0.407 |
| Ornithine | 0.08 ± 0.01 | 0.08 ± 0.01 | | 0.07 ± 0.01 | 0.08 ± 0.01 | 0.458 | 0.415 | 0.525 |
| Taurine | 1.71 ± 0.10 | 1.73 ± 0.08 | | 1.80 ± 0.08 | 1.81 ± 0.08 | 0.133 | 0.831 | 0.877 |

^1^ Units: µmol/g fresh tissue weight. Values are raw means ± SE; n = 12 per BiW x Supplementation (Suppl) group. Interaction is the BiW x Suppl interaction.

^2^ Abbreviations: Aad, alpha-aminoadipic acid; Abu, alpha-aminobutyric acid; Aib, α-aminoisobutyric acid; GABA, gamma-aminobutyric Acid; HP, hydroxyproline.

^3^ ANOVA *F* Test.

^a,b^ Different from Alanine-supplemented piglets within BiW group (*P* < 0.05).

^c,d^ Different from NBiW piglets within supplementation group (*P* < 0.05).
