# Supplementary material for: Relationship between neonatal glutamine supplementation and amino acid profiles, protein synthesis in splanchnic tissues and the plasma metabolome in 5-day-old piglets with low birth weight
Source: PLoS One. 2026 Oct 12;21(10):e0359788. doi: 10.1371/journal.pone.0359788 (PMC13657303; doi:10.1371/journal.pone.0359788)
Supplement: Supplementary file 9 [file pone.0359788.s009.docx]

**S8 Table.** **Duodenal tissue protein-bound amino acid profiles in low (LBiW) and normal (NBiW) birthweight (BiW) piglets supplemented with glutamine or alanine from 24 h after birth to 5 d of age.**

| **Item^1,2^** | **LBiW** | | **NBiW** | | | ***P*-value^3^** | | |
| --- | --- | --- | --- | --- | --- | --- | --- | --- |
|  | **Alanine** | **Glutamine** | | **Alanine** | **Glutamine** | **BiW** | **Suppl** | **Interaction** |
| **AA** |  |  | |  |  |  |  |  |
| **Indispensable AA** | 280.25 ± 9.66 | 275.58 ± 9.72 | | 281.47 ± 9.87 | 259.51 ± 10.06 | 0.311 | 0.123 | 0.239 |
| Arginine | 32.29 ± 1.47 | 31.54 ± 1.47 | | 32.78 ± 1.49^a^ | 29.64 ± 1.47^b^ | 0.407 | 0.073 | 0.167 |
| Histidine | 11.68 ± 0.42 | 11.50 ± 0.43 | | 11.53 ± 0.43 | 10.76 ± 0.43 | 0.168 | 0.209 | 0.354 |
| Isoleucine | 30.54 ± 1.00 | 29.87 ± 1.01 | | 30.48 ± 1.02^a^ | 28.04 ± 1.04^b^ | 0.218 | 0.084 | 0.248 |
| Leucine | 56.03 ± 1.91 | 54.81 ± 1.92 | | 56.22 ± 1.95 | 51.99 ± 1.99 | 0.361 | 0.109 | 0.296 |
| Lysine | 40.62 ± 1.68 | 40.29 ± 1.69 | | 41.33 ± 1.71 | 37.83 ± 1.73 | 0.433 | 0.158 | 0.161 |
| Methionine | 12.53 ± 0.46 | 12.23 ± 0.46 | | 12.54 ± 0.47 | 11.60 ± 0.48 | 0.347 | 0.117 | 0.335 |
| Phenylalanine | 22.64 ± 0.81 | 22.46 ± 0.82 | | 22.94 ± 0.83 | 21.01 ± 0.84 | 0.351 | 0.146 | 0.161 |
| Threonine | 29.70 ± 1.30 | 29.82 ± 1.31 | | 29.41 ± 1.33 | 27.06 ± 1.31 | 0.101 | 0.308 | 0.178 |
| Tryptophan | 5.77 ± 0.21 | 5.75 ± 0.21 | | 5.75 ± 0.22^a^ | 5.23 ± 0.21^b^ | 0.097 | 0.155 | 0.124 |
| Valine | 37.63 ± 1.30 | 37.29 ± 1.31 | | 37.70 ± 1.32^a^ | 34.43 ± 1.31^b^ | 0.150 | 0.116 | 0.130 |
| **Dispensable AA** | 283.77 ± 11.74 | 284.27 ± 11.84 | | 281.89 ± 12.00 | 260.45 ± 11.84 | 0.151 | 0.316 | 0.218 |
| Alanine | 44.67 ± 1.63 | 43.15 ± 1.64 | | 44.18 ± 1.66^a^ | 40.04 ± 1.64^b^ | 0.144 | 0.055 | 0.281 |
| Asparagine | 20.75 ± 0.91 | 21.02 ± 0.92 | | 20.58 ± 0.93 | 19.04 ± 0.92 | 0.113 | 0.423 | 0.178 |
| Aspartate | 27.76 ± 1.97 | 28.66 ± 1.97 | | 26.91 ± 2.00 | 25.78 ± 1.97 | 0.138 | 0.937 | 0.413 |
| Cysteine | 3.38 ± 0.21 | 3.27 ± 0.21 | | 3.56 ± 0.21 | 3.35 ± 0.21 | 0.423 | 0.384 | 0.766 |
| Glutamate | 41.76 ± 2.59 | 44.22 ± 2.60 | | 40.18 ± 2.64 | 40.10 ± 2.60 | 0.115 | 0.576 | 0.475 |
| Glutamine | 25.48 ± 0.96 | 23.38 ± 0.97 | | 25.52 ± 0.98^a^ | 22.30 ± 0.97^b^ | 0.466 | 0.003 | 0.434 |
| Glycine | 42.51 ± 2.07 | 42.38 ± 2.08 | | 41.94 ± 2.11 | 38.36 ± 2.08 | 0.086 | 0.251 | 0.191 |
| Proline | 22.64 ± 1.62 | 25.47 ± 1.63 | | 24.19 ± 1.65 | 22.53 ± 1.63 | 0.525 | 0.659 | 0.048 |
| Serine | 36.55 ± 1.39 | 35.87 ± 1.40 | | 36.15 ± 1.42 | 33.36 ± 1.40 | 0.170 | 0.162 | 0.314 |
| Tyrosine | 17.45 ± 0.69 | 17.36 ± 0.70 | | 17.72 ± 0.71^a^ | 16.09 ± 0.70^b^ | 0.315 | 0.153 | 0.125 |
| **Total AA** | 559.28 ± 20.82 | 556.27 ± 20.99 | | 558.52 ± 21.27 | 511.89 ± 20.99 | 0.150 | 0.179 | 0.164 |
| **BCAA** | 124.44 ± 4.12 | 121.93 ± 4.15 | | 124.64 ± 4.21 | 115.07 ± 4.30 | 0.294 | 0.106 | 0.266 |

^1^ Units: µmol/g fresh tissue weight. Values are LSM ± SE; n = 12 per BiW x Supplementation (Suppl) group. Interaction is the BiW x Suppl interaction.

^2^ Abbreviations: AA, amino acids; BCAA, branched chain AA.

^3^ ANOVA *F* Test.

^a,b^ Different from Alanine-supplemented piglets within BiW group (*P* < 0.05).

^c,d^ Different from NBiW piglets within supplementation group (*P* < 0.05).
