# Supplementary material for: Relationship between neonatal glutamine supplementation and amino acid profiles, protein synthesis in splanchnic tissues and the plasma metabolome in 5-day-old piglets with low birth weight
Source: PLoS One. 2026 Oct 12;21(10):e0359788. doi: 10.1371/journal.pone.0359788 (PMC13657303; doi:10.1371/journal.pone.0359788)
Supplement: Supplementary file 10 [file pone.0359788.s010.docx]

**S9 Table. Duodenal mRNA abundance in low (LBiW) and normal (NBiW) birthweight (BiW) piglets supplemented with glutamine or alanine from 24 h after birth to 5 d of age.**

| **Item^1^** | **LBiW** | | | | **NBiW-Ala** | | | ***P*-values^2^** | | |
| --- | --- | --- | --- | --- | --- | --- | --- | --- | --- | --- |
|  | **Alanine** | | **Glutamine** | | **Alanine** | | **Glutamine** | **BiW** | **Suppl** | **Interaction** |
| **Amino acid transport** | |  | |  | |  | |  |  |  |
| SLC6A19 | 0.02 ± 0.06^a^ | | -0.15 ± 0.06^b,d^ | | 0.04 ± 0.07 | | 0.05 ± 0.06^c^ | 0.042 | 0.180 | 0.111 |
| SLC7A8 | 0.01 ± 0.07^a^ | | -0.21 ± 0.07^b,d^ | | 0.03 ± 0.07 | | 0.03 ± 0.07^c^ | 0.227 | 0.037 | 0.041 |
| **Glutamine metabolism** | |  | |  | |  | |  |  |  |
| AST1 | 0.03 ± 0.06 | | -0.11 ± 0.05^d^ | | 0.06 ± 0.06 | | 0.03 ± 0.05^c^ | 0.079 | 0.132 | 0.225 |
| AST2 | -0.004 ± 0.03 | | 0.001 ± 0.03 | | 0.02 ± 0.03 | | -0.01 ± 0.03 | 0.849 | 0.597 | 0.446 |
| GLS | 0.02 ± 0.04 | | 0.01 ± 0.05 | | 0.04 ± 0.04 | | -0.03 ± 0.04 | 0.880 | 0.335 | 0.397 |
| GLUD1 | 0.02 ± 0.03 | | 0.01 ± 0.03 | | -0.03 ± 0.03 | | -0.02 ± 0.03 | 0.127 | 0.962 | 0.556 |
| NAGS | -0.05 ± 0.04 | | -0.02 ± 0.04 | | 0.05 ± 0.04 | | 0.03 ± 0.04 | 0.085 | 0.926 | 0.582 |
| **Glutathione synthesis** | |  | |  | |  | |  |  |  |
| GCLC | -0.02 ± 0.03 | | 0.03 ± 0.03 | | -0.01 ± 0.03 | | 0.001 ± 0.03 | 0.718 | 0.371 | 0.553 |
| CGL | 0.02 ± 0.03 | | -0.04 ± 0.03 | | 0.03 ± 0.03 | | 0.01 ± 0.03 | 0.174 | 0.210 | 0.431 |
| GGT | -0.02 ± 0.04 | | 0.05 ± 0.04 | | 0.07 ± 0.04 | | 0.02 ± 0.04 | 0.488 | 0.870 | 0.122 |
| GPX1 | 0.01 ± 0.04 | | -0.008 ± 0.039 | | 0.02 ± 0.04 | | 0.03 ± 0.04 | 0.421 | 0.860 | 0.683 |
| GPX2 | -0.08 ± 0.11 | | 0.17 ± 0.12 | | -0.06 ± 0.12 | | -0.07 ± 0.11 | 0.361 | 0.293 | 0.260 |
| GPX4 | -0.003 ± 0.033 | | 0.01 ± 0.03 | | 0.02 ± 0.03 | | -0.02 ± 0.03 | 0.870 | 0.714 | 0.314 |
| GSR | -0.02 ± 0.02 | | 0.03 ± 0.02 | | 0.01 ± 0.02 | | -0.004 ± 0.018 | 0.665 | 0.328 | 0.070 |
| GSS | -0.04 ± 0.02^b^ | | 0.04 ± 0.02^a^ | | 0.005 ± 0.023 | | 0.01 ± 0.02 | 0.522 | 0.041 | 0.067 |
| GSTA1 | 0.06 ± 0.06 | | 0.14 ± 0.06 | | -0.02 ± 0.06 | | 0.01 ± 0.06 | 0.051 | 0.331 | 0.607 |
| OPLAH | -0.06 ± 0.05^d^ | | 0.004 ± 0.050 | | 0.09 ± 0.05^c^ | | -0.01 ± 0.05 | 0.165 | 0.675 | 0.093 |
| **Tight junction proteins** | |  | |  | |  | |  |  |  |
| TJP1 | 0.03 ± 0.02^a^ | | -0.04 ± 0.02^b^ | | 0.02 ± 0.02 | | -0.03 ± 0.02 | 0.799 | 0.004 | 0.487 |
| TJP2 | 0.02 ± 0.03 | | 0.002 ± 0.029 | | 0.06 ± 0.03 | | 0.02 ± 0.03 | 0.272 | 0.275 | 0.559 |
| CLDN4 | -0.03 ± 0.06 | | -0.01 ± 0.06 | | 0.06 ± 0.06 | | -0.03 ± 0.06 | 0.358 | 0.463 | 0.200 |
| **Miscellanous** | |  | |  | |  | |  |  |  |
| CASP3 | 0.08 ± 0.04^a^ | | -0.05 ± 0.04^b^ | | 0.02 ± 0.04 | | -0.01 ± 0.04 | 0.678 | 0.055 | 0.136 |
| SDHA | -0.01 ± 0.03 | | -0.03 ± 0.03 | | 0.03 ± 0.03 | | 0.005 ± 0.03 | 0.130 | 0.443 | 0.889 |
| SOD1 | -0.01 ± 0.02 | | 0.01 ± 0.02 | | 0.02 ± 0.02 | | 0.01 ± 0.02 | 0.438 | 0.667 | 0.390 |

^1^ Units: log-CNRQ. Values are LSM ± SE; n = 12 per BiW x Supplementation (Suppl) group. Interaction is the BiW x Suppl interaction.

^2^ ANOVA *F* Test.

^a,b^ Different from Alanine-supplemented piglets within BiW group (*P* < 0.05).

^c,d^ Different from NBiW piglets within supplementation group (*P* < 0.05).
