# Supplementary material for: Relationship between neonatal glutamine supplementation and amino acid profiles, protein synthesis in splanchnic tissues and the plasma metabolome in 5-day-old piglets with low birth weight
Source: PLoS One. 2026 Oct 12;21(10):e0359788. doi: 10.1371/journal.pone.0359788 (PMC13657303; doi:10.1371/journal.pone.0359788)
Supplement: Supplementary file 11 [file pone.0359788.s011.docx]

**S10 Table. Liver, duodenum and colon biochemical indices and fractional protein synthesis rate (FPSR) in low (LBiW) and normal (NBiW) birthweight (BiW) piglets supplemented with glutamine or alanine from 24 h after birth to 5 d of age.**

| **Item^1^** | **LBiW** | | **NBiW** | | | ***P*-values^2^** | | |
| --- | --- | --- | --- | --- | --- | --- | --- | --- |
|  | **Alanine** | **Glutamine** | | **Alanine** | **Glutamine** | **BiW** | **Suppl** | **Interaction** |
| **Liver** |  |  | |  |  |  |  |  |
| DNA | 2.49 ± 0.22 | 2.63 ± 0.21 | | 2.42 ± 0.22 | 2.48 ± 0.22 | 0.492 | 0.621 | 0.822 |
| RNA | 3.82 ± 0.39 | 4.05 ± 0.38 | | 4.63 ± 0.39 | 4.02 ± 0.38 | 0.258 | 0.613 | 0.218 |
| Protein | 96.38 ± 2.97^b^ | 103.04 ± 2.90^a^ | | 99.38 ± 3.26 | 100.90 ± 2.90 | 0.845 | 0.126 | 0.246 |
| Protein to DNA ratio | 45.33 ± 4.26 | 40.86 ± 4.27 | | 44.90 ± 4.26 | 38.09 ± 4.86 | 0.650 | 0.188 | 0.739 |
| Protein to RNA ratio | 29.43 ± 3.16^c^ | 29.65 ± 3.09 | | 21.12 ± 3.29^d^ | 25.11 ± 3.22 | 0.029 | 0.503 | 0.507 |
| RNA to DNA ratio | 1.49 ± 0.20^d^ | 1.60 ± 0.19 | | 1.95 ± 0.20^c^ | 1.54 ± 0.19 | 0.223 | 0.411 | 0.110 |
| FPSR | 96.75 ± 8.81 | 105.47 ± 9.24 | | 94.68 ± 8.44 | 94.54 ± 8.44 | 0.463 | 0.628 | 0.617 |
| **Duodenum^3^** |  |  | |  |  |  |  |  |
| RNA | 3.27 ± 0.16^a^ | 2.71 ± 0.16^b^ | | 2.90 ± 0.16 | 2.62 ± 0.15 | 0.152 | 0.012 | 0.354 |
| Protein | 86.79 ± 1.72 | 84.44 ± 1.56 | | 88.10 ± 1.72 | 84.3 ± 1.68 | 0.648 | 0.046 | 0.588 |
| FPSR | 62.85 ± 8.50 | 68.04 ± 7.95 | | 62.36 ± 6.78 | 61.31 ± 6.78 | 0.638 | 0.787 | 0.684 |
| **Colon** |  |  | |  |  |  |  |  |
| FPSR | 29.41 ± 3.72^b^ | 34.77 ± 3.48 | | 40.21 ± 3.11^a^ | 37.75 ± 3.72 | 0.068 | 0.685 | 0.283 |

^1^ Units: DNA, RNA and Protein (µg / mg Tissue); FPSR (% d-1). Values are LSM ± SE; LBiW-Ala, *n* = 7-12; LBiW-Gln, *n* = 8-14; NBiW-Ala, *n* = 9-12; NBiW-Gln, *n* = 7-12. Interaction is the BiW x Supplementation (Suppl) interaction.

^2^ ANOVA *F* Test.

^3^ Not enough duodenum mucosa sample remained after RNA extraction to extract DNA.

^a,b^ Different from Alanine-supplemented piglets within BiW group (*P* < 0.05); ^c,d^ Different from NBiW piglets within supplementation group (*P* < 0.05).
